# Selective overexpression of cytoglobin in stellate cells attenuates thioacetamide-induced liver fibrosis in mice

**DOI:** 10.1038/s41598-018-36215-4

**Published:** 2018-12-14

**Authors:** Nguyen Thi Thanh Hai, Le Thi Thanh Thuy, Akira Shiota, Chiho Kadono, Atsuko Daikoku, Dinh Viet Hoang, Ninh Quoc Dat, Misako Sato-Matsubara, Katsutoshi Yoshizato, Norifumi Kawada

**Affiliations:** 1 Departments of Hepatology, Graduate School of Medicine, Osaka City University, Osaka, Japan; 2 Department of Biochemistry, Hanoi Medical University, Hanoi, Vietnam; 3 PhoenixBio Co. Ltd., Hiroshima, Japan; 4 Endowed Laboratory of Synthetic Biology, Graduate School of Medicine, Osaka City University, Osaka, Japan

## Abstract

Cytoglobin (CYGB), discovered in hepatic stellate cells (HSCs), is known to possess a radical scavenger function, but its pathophysiological roles remain unclear. Here, for the first time, we generated a new transgenic (TG) mouse line in which both Cygb and mCherry reporter gene expression were under the control of the native *Cygb* gene promoter. We demonstrated that the expression of Cygb-mCherry was related to endogenous *Cygb* in adult tissues by tracing mCherry fluorescence together with DNA, mRNA, and protein analyses. Administration of a single dose (50 mg/kg) of thioacetamide (TAA) in Cygb-TG mice resulted in lower levels of alanine transaminase and oxidative stress than those in WT mice. After 10 weeks of TAA administration, Cygb-TG livers exhibited reduced neutrophil accumulation, cytokine expression and fibrosis but high levels of quiescent HSCs. Primary HSCs isolated from Cygb-TG mice (HSC^Cygb-TG^) exhibited significantly decreased mRNA levels of α-smooth muscle actin (αSMA), collagen 1α1, and transforming growth factor β-3 after 4 days in culture relative to WT cells. HSCs^Cygb-TG^ were resistant to H_2_O_2_-induced αSMA expression. Thus, cell-specific overexpression of Cygb attenuates HSC activation and protects mice against TAA-induced liver fibrosis presumably by maintaining HSC quiescence. Cygb is a potential new target for antifibrotic approaches.

## Introduction

Cytoglobin (CYGB) is one of the five recognized globins, including haemoglobin (HB), myoglobin (MB), neuroglobin (NGB), and androglobin (ADB), in vertebrates^1,2^. CYGB was originally discovered in rat hepatic stellate cells (HSCs) and named stellate cell activation-associated protein (STAP)^3^, but it was renamed cytoglobin given its localization in the cytoplasm and globin-like structure^1,4,5^. Human *CYGB* displays ~25% amino acid identity with vertebrate MB and HB and 16% identity with human NGB. The *CYGB* gene is localized to chromosome 17q25.3 in humans and chromosome 11E2 in mice^4,6–8^.

Unlike other globins, which show tissue-restricted expression patterns, such as HB in erythrocytes, MB in cardiomyocytes and skeletal myofibers, NGB in the nervous system, and ADB in testis^2^, CYGB is ubiquitously expressed in the cytoplasm of mesenchymal fibroblastic cells in many organs, including the brain, heart, lung, liver, kidney, intestine, and spleen^1,9^. The presence of CYGB in the nuclei of these cells has also been reported^9,10^. In particular, CYGB was shown to be present in stellate cells and myofibroblasts in the liver and pancreas, reticulocytes in the spleen, mesenchymal cells in the submucosal layer of the gut, and the mesangium and stromal cells of the kidney^9^.

Cygb exhibits intrinsic oxygen (O_2_) -binding capacity; its haem iron was demonstrated similar affinities for exogenous ligands and equilibrium constants for O_2_ as those observed in Mb^3,11^. Regarding its distribution in fibroblast-like cells, which are not generally associated with high metabolic rates and oxygen consumption, CYGB might act as an oxygen sensor and be involved in cell proliferation and possibly oxygen diffusion for collagen synthesis^12^. Recently, our group demonstrated that CYGB in (HSCs) has a role in augmenting the O_2_ supply to hepatocytes during cytochrome P450-mediated xenobiotic oxidative metabolism induced by acetaminophen or carbon tetrachloride (CCl_4_) treatment^13^. Since O_2_ binding imposes conformational changes on the disulphide bridge, a shift in CYGB structure and concomitant O_2_ release^14^, CYGB may putatively act as a signal transducer of the pathways associated with oxygen sensing^11,15^.

Liver injury triggers HSC activation, which has been identified as a key event in hepatic fibrogenesis. During the activation process, HSCs are known to acquire proliferative, fibrogenic and contractile activities^16^. Since CYGB was initially found to be induced in activated HSCs^3^, the expression of CYGB was suggested to protect HSCs during liver injury when they are increasingly exposed to both endogenous and exogenous reactive oxygen species (ROS). The ROS scavenging function of CYGB, therefore, could be illuminated, as evidenced by its ability to detoxify radicals via reaction with its haeme^11,17,18^. Similarly, forced overexpression of CYGB was reported to significantly augment the total oxyradical scavenging capacity relative to the expression of eGFP^19^.

In our previous studies, administration of the well-known carcinogen diethynitrosamine (DEN)^20^, a choline-deficient, L-amino acid-defined (CDAA) diet^21^, or bile duct ligation^22^ induced dominant liver fibrosis in *Cygb* knockout (*Cygb*^−/−^) mice relative to wild-type mice (WT). Cytologically, HSCs in the absence of *Cygb* (HSCs^*Cygb*-null^) became enlarged with a developed α-smooth muscle actin (αSMA) network after 7 days in culture, and these cells lost cellular vitamin A-lipid droplets more rapidly than HSCs from WT mice (HSCs^*Cygb-*WT^)^21^. Moreover, HSCs^*Cygb*-null^ demonstrated a pre-activated phenotype with increased oxidative stress and strong expression of cytokines and chemokines, such as interleukin 6 (*Il-6*), tumour necrosis factor (*Tnf*) α, interleukin 1β (*Il-1β*), chemokine (C-X-C motif) ligand 1 (*Cxcl-1*), *Cxcl-2*, and chemokine (C-C motif) ligand 2 (*Ccl-2*), -3, and -4^21^. Taken together, *Cygb* deficiency promotes fibrosis development, likely via HSC activation.

In the present study, we generated a transgenic (TG) mouse line to drive Cygb gene and mCherry reporter gene expression under the control of the native *Cygb* gene promoter *in vivo*. This transgenic mouse lineage stably transmitted 10 copies of the BAC transgenic locus. By using these cell -type- specific Cygb-TG mice in a hepatic injury and fibrosis model, we demonstrated that Cygb overexpression attenuated collagen deposition in the liver, possibly by inhibiting HSC activation through augmented ROS scavenger function.

## Results

### Generation and characterization of Cygb-2A-mCherry BAC transgenic mice

**Figure 1:**
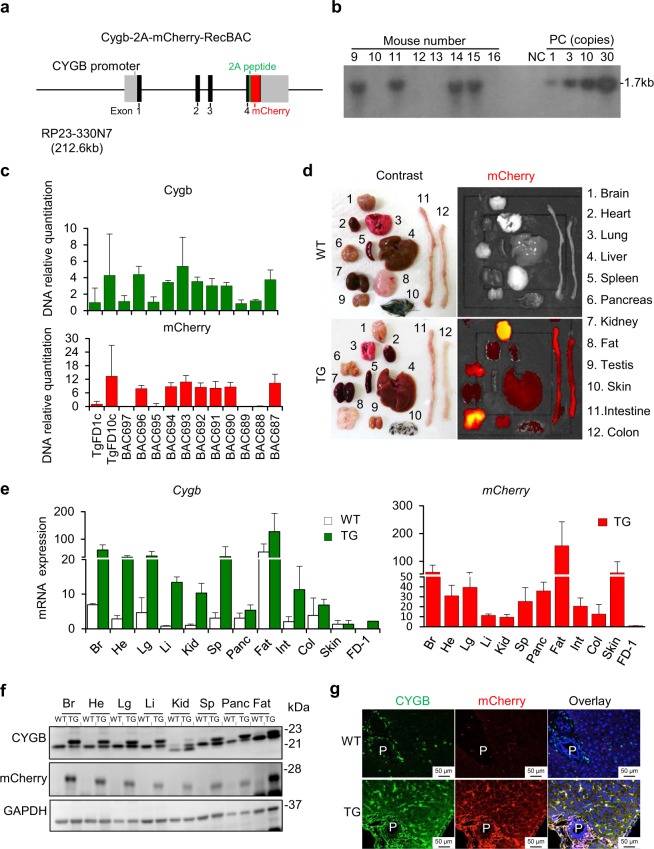
Generation of hepatic stellate cell-specific Cygb-transgenic mice. (**a**) DNA construct map for Cygb-mCherry. The Cygb-2A-mCherry reporter was generated with the BAC clone RP23-330N7A, and a partial genomic map of the *Cygb* gene with coding exons (black boxes), noncoding regions including the promoter of the *Cygb* gene (light grey boxes), and flanking introns (solid lines) is shown. The 2A-mCherry reporter gene, which was flanked by 110 bp of the upstream sequence of the Cygb gene stop codon and 87 bp of the downstream sequence of its stop codon, was precisely transferred to the Cygb gene. (**b**) Genotyping of Cygb-transgenic **(**TG) mice in the F1 generation by Southern blots showed TG mice (lanes 9, 11, 14, and 15) bearing 10 copies of the Cygb transgenes. NC, DNA negative control; PC, DNA positive controls with 1, 3, 10, and 30 copies. (**c**) Genotyping of offspring by real-time qRT-PCR. DNA isolated from tail biopsies of Cygb 10 copies-transgenic founder (TgFD10c) mice was used as a positive control, and DNA from the 1-copy founder (TgFD1c) was used as a reference sample. Relative quantification of Cygb (green bars) and mCherry (red bars) DNA is shown. Mouse numbers BAC 687, 690–694, and 696 were clarified as Cygb-TG, and the remaining mice were WT. The relative number of DNA copies was normalized to *Gapdh* levels. (**d**) Macroscopic view of multiple organs of WT and Cygb-TG mice under a contrast photo (right panel) and fluorescence images of Cherry (left panel). (**e**) Real-time qRT-PCR analysis shows the *Cygb* expression levels in multiple organs of WT (white bars) and TG mice (green bars). Transcriptional levels of only mCherry were examined in TG mice (red bars). *Gapdh* was used as an endogenous control. (**f**) Immunoblot analysis showed the CYGB and mCherry protein levels in multiple organs of WT and TG mice. GAPDH was used as the loading control and for normalization. Full-length Western blots in one gel are presented in Supplementary Fig. S6. (**g**) Representative imaging of CYGB (green) and mCherry (red) immunofluorescence staining from WT and TG livers. Br, Brain; Li, Liver; Panc, Pancreas; He, Heart; Lg, Lung; Int, Intestine; Sp, Spleen; Kid, Kidney. P, portal vein; C, central vein.

To develop a mouse model for tracing *Cygb*-expressing cells and their role in whole organs, we generated a transgenic mouse lines to drive Cygb and mCherry reporter gene expression under the control of the native *Cygb* gene *in vivo*. Bacterial artificial chromosome (BAC) transgenesis is a powerful tool for transgenic expression that is copy -number- dependent and integration -position- independent^23,24^. To this end, we identified a Cygb BAC clone that included approximately 80 kb upstream of the transcription initiation codon, the entire Cygb structural gene, and the region downstream of the termination codon of the gene. Relying on the ability of the Cygb genomic sequences to correctly target gene expression, we precisely removed the stop codon sequence of the Cygb gene from its BAC clone and transferred the 2A-mCherry gene by BAC recombineering. The chimeric Cygb-2A-mCherry BAC transgenic construct abundantly expressed *Cygb* and *mCherry* mRNA via the 2 A peptide bridge within HSCs in the liver and pericytes in other organs of Cygb-TG mice (Fig. 1a).

Following BAC modification in *E. coli*, the linearized BAC transgenic construct was purified and injected into pronucleus-stage mouse embryos to generate independent transgenic founders. By breeding, the founder stably transmitted 10 copies of the BAC transgenic locus to F1 pups, which was confirmed by Southern blots, as shown in Fig. 1b. This transgenic mouse lineage was used for breeding with WT counterparts, heterozygote crosses, to maintain Cygb-TG mice and for the following experiments.

The DNA samples examined for Southern blotting were used as positive controls for routine genotyping of Cygb-TG mice by quantitative real-time PCR for both Cygb and mCherry expression using DNA isolated from tail-cut samples (Fig. 1c). Analysis of the tail biopsy specimens at the age of 6 weeks revealed the presence of Cygb-TG mice at a frequency of 43% among 747 offspring of heterozygote crosses. Body weight, blood pressure, and biochemical analysis of blood samples showed negligible differences between the Cygb-TG mice and their WT littermates, except for high density lipoprotein cholesterol (HDL-C) serum level (Supplementary Fig. S1a,b & Supplementary Table S1-2), and no specific phenotypic changes were observed in Cygb-TG mice from 4 weeks until 24 months old. Baseline histological analysis of Cygb-TG livers by H&E staining showed that their morphology was similar to that of WT livers (Supplementary Fig. S1c).

The expression of Cygb-mCherry under the endogenous *Cygb* promoter in adult tissues, including the brain, heart, lung, thymus, liver, spleen, kidney, pancreas, intestine, colon, skin, and fat, was live- imaged using an *in vivo* fluorescence imaging system (IVIS). Representative images of red fluorescence signals of mCherry at a wavelength of 610 nm are shown in Fig. 1d, but this signal was absent in WT samples.

mRNA expression levels in all tissues from Cygb-TG mice relative to those in WT mice are shown in Fig. 1e. Compared to the founder mouse with 1 copy of the Cygb transgene, Cygb-TG mice had 10 copies of the Cygb gene in almost all organs and showed more than ten-fold increases in the transcription levels of both *Cygb* and *mCherry* mRNA. Of note, *Cygb* expression was most abundant in the fat, brain, spleen and lungs (Fig. 1e).

At the protein level, immunoblot analyses showed the expression of CYGB and mCherry in paired tissues in WT and Cygb-TG mice. In WT samples, there was a single band of the CYGB protein at 21 (kDa) (Fig. 1f). In Cygb-TG samples, both the endogenous CYGB protein at 21 kDa (lower bands) and the transgene 2A-linked-CYGB protein at 23 kDa (upper bands) were observed. mCherry expression at 28 kDa was found only in Cygb-TG mice (Fig. 1f).

To visualize the local expression of CYGB and mCherry, we performed double immunofluorescence staining in liver and all tissues. The results showed that CYGB and mCherry co-localized in the cytoplasm of pericytes of all organs (Supplementary Fig. S2). In the liver, co-localization of CYGB and mCherry was found in HSCs along the hepatic sinusoid (Fig. 1g). mCherry was also co-localized with desmin, a marker of HSCs (Supplementary Fig. S3a), but not with CD31, a marker of endothelial cells (Supplementary Fig. S3b), by double immunofluorescence staining.

### Overexpression of Cygb attenuated hepatic stellate cell activation

**Figure 2:**
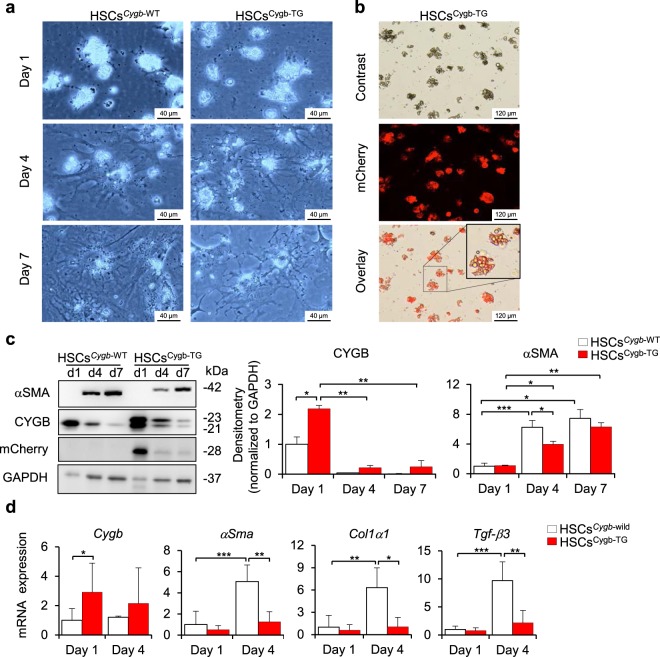
Overexpression of Cygb attenuates HSC activation. (**a**) Morphology of primary hepatic stellate cells from WT (HSCs^*Cygb-*WT^) and TG mice (HSCs^Cygb-TG^) at days 1, 4 and 7. (**b**) Image of lipid droplets and fluorescent mCherry protein in primary HSCs^Cygb-TG^ at day 1. Inset, 3x from original pictures. (**c**) Immunoblot analysis of CYGB, mCherry and αSMA protein expression in primary HSCs^*Cygb-*WT^ and HSCs^Cygb-TG^ under normal conditions. Right panels show the quantification of the densitometric intensity. Levels were normalized to GAPDH. Full-length Western blots in one gel are presented in Supplementary Fig. S7. (**d**) mRNA expression levels of *Cygb*, *αSma*, *Col1α1* and *Tgf-β3* in primary HSCs^*Cygb-*WT^ (white bars) and HSCs^Cygb-TG^ (red bars) at days 1 and 4 were determined by RT-qPCR (n = 5 to 10). Levels were normalized to *Gapdh*. Values are given as the mean ± SD of all experiments. *p ≤ 0.05, **p ≤ 0.01, ***p ≤ 0.001.

Next, we focused our efforts on examining the phenotype of HSCs in Cygb-TG mice since CYGB is uniquely expressed in HSCs. Primary HSCs isolated from WT (HSCs^*Cygb*-WT^) and Cygb-TG mice (HSCs^Cygb-TG^) were cultured in 10% FBS/DMEM, and we observed the phenotype from day 1 to day 7. At day 1, HSCs^*Cygb*-WT^ were rounded and contained multiple lipid droplets in the cytoplasm; from day 4 to day 7, they gradually lost cytoplasmic lipid droplets and displayed branching with angular and long cytoplasmic membranous processes decorated with numerous spines (Fig. 2a, left panels). In contrast, HSCs^Cygb-TG^ were less enlarged than WT cells (Fig. 2a, right panels). Under fluorescence microscopy, we observed mCherry expression around lipid droplets in the cytoplasm of HSCs (Fig. 2b). By immunoblot analysis, we confirmed that CYGB protein expression showed a 2.5-fold increase in HSCs^Cygb-TG^ relative to HSCs^*Cygb*-WT^ (p < 0.05) at day 1. Notably, CYGB protein expression was significantly downregulated from day 1 to day 7 in culture, while αSMA, a marker of activated HSCs, was increased in both HSCs^*Cygb-*WT^ and HSCs^Cygb-TG^. However, protein expression of αSMA was significantly suppressed in HSCs^Cygb-TG^ at day 4 relative to HSCs^*Cygb-*WT^ (Fig. 2c). HSCs^Cygb-TG^ exhibited decreased expression of fibrosis-related genes at the mRNA level, including *αSma* (4-fold lower, p < 0.01), collagen 1 anpha 1 (*Col1α1)* (6-fold lower, p < 0.05), and transforming growth factor beta 3 (*Tgfβ-*3) (4.5-fold lower, p < 0.01), after 4 days of culture relative to HSCs^*Cygb*-WT^ (Fig. 2d). These results indicate that overexpression of Cygb attenuates HSC activation.

### Attenuation of liver injury and oxidative stress during the acute phase of thioacetamide treatment in Cygb-TG mice

**Figure 3:**
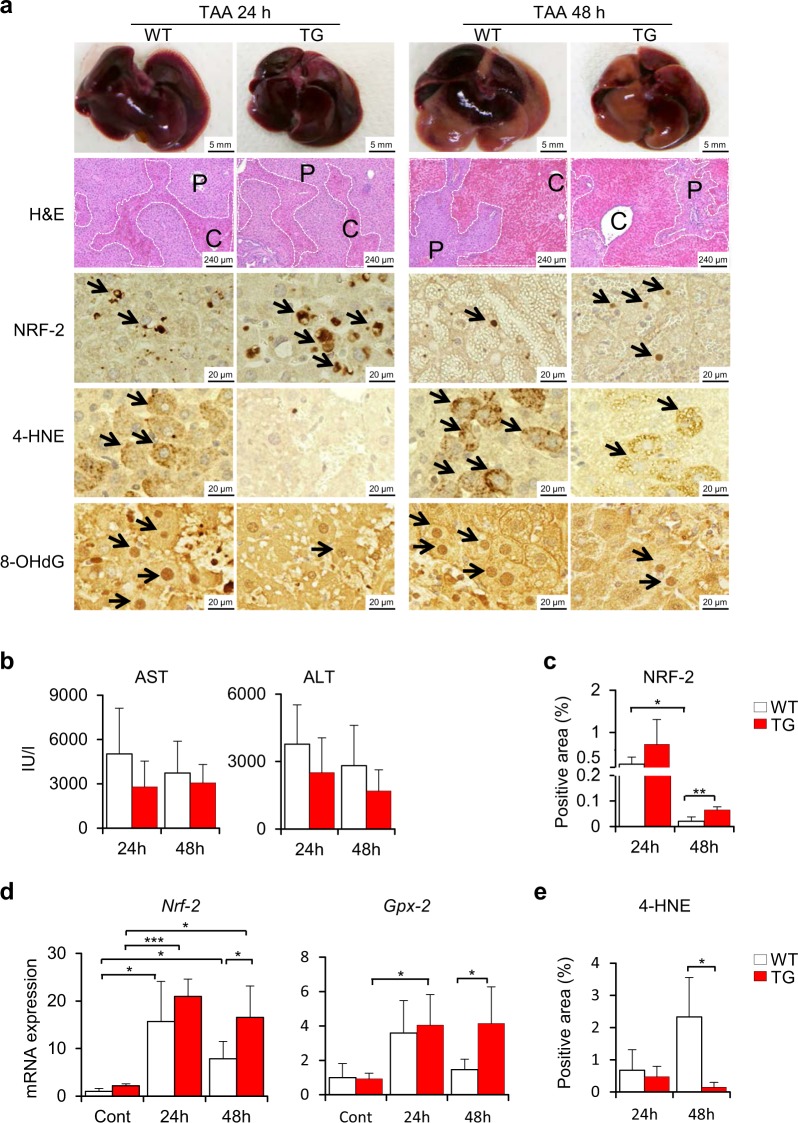
Suppression of oxidative stress in Cygb-TG mice challenged with a single dose of TAA. (**a**) Macroscopic and microscopic view of liver injuries in wild- type (WT) and Cygb-TG mice (TG) after 24 hours (24 h) or 48 hours (48 h) of exposure to a single dose of TAA. Representative images of gross appearance, H&E staining, and immunohistochemistry staining for erythroid 2–related factor 2 (NRF-2), 4-hydroxynonenal (4-HNE), and 8-hydroxy-2′-deoxyguanosine (8-OHdG). P, portal vein; C, central vein. Dashed line shows the haemorrhagic area. (**b**) Plasma levels of AST and ALT were measured. (**c**) Percentages of NRF-2-positive areas of liver sections were quantified. (**d**) Transcription of *Nrf-2* - the antioxidative signalling pathway and its downstream component - glutathione peroxidase 2 (*Gpx-2*). (**e**) Percentages of 4-HNE-positive areas per 20 random micro fields in liver sections at 400x magnification were quantified. Data are expressed as the mean ± SD (n = 5), *p ≤ 0.05, **p ≤ 0.01.

To ascertain the role of *Cygb* in liver pathophysiology, we challenged Cygb-TG and WT mice with acute-toxic liver injury induced by the administration of thioacetamide (TAA), which is known to cause membrane damage and oxidative stress in the hepatocyte cytoplasm^25^. After intraperitoneal (*i.p*.) administration of one dose of TAA (50 mg/kg body weight), macroscopic views and haematoxylin & eosin (H&E) staining of Cygb-TG and WT livers showed the occurrence of hepatic haemorrhage in both mouse strains at both 24 and 48 h (Fig. 3a). H&E staining showed that the hepatic haemorrhage area was approximately equal between Cygb-TG and WT samples (Fig. 3a and Supplementary Fig. S4). Levels of serum alanine aminotransferase (ALT) and aspartate aminotransferase (AST) were slightly lower in Cygb-TG mice than WT mice, although this difference was not significant (Fig. 3b).

Given that TAA induces the apoptosis and necrosis of hepatocytes mainly via its metabolites TAA sulfoxide (TASO) and TAA-S,S dioxide (TASO_2_) and by the generation of reactive oxygen species (ROS)^26^, we assessed the oxidative stress response in these mice. Both the mRNA and protein levels of nuclear factor erythroid 2–related factor 2 (NRF-2), an emerging antioxidant regulator of cellular resistance to oxidants^27^, were strongly induced in TAA-treated TG livers at 24 h and then decreased at 48 h but still remained higher than those of the WT samples (3-fold higher protein level at 48 h, p < 0.01) (Fig. 3a,c, and d). Consistent with this result, one of the *Nrf-2* target genes, glutathione peroxidase 2 (*Gpx-2*), was transcriptionally increased in TAA-treated Cygb-TG mice (3-fold higher, p < 0.05) relative to WT mice at 48 h (Fig. 3d). Both 4-hydroxynonenal (4-HNE), a lipid peroxidation product, and 8-hydroxy-2′-deoxyguanosine (8-OHdG), a product of oxidatively damaged DNA formed by hydroxyl radicals, singlet oxygen and direct photodynamic action, were abundant in TAA-treated WT livers but suppressed in Cygb-TG livers (4-HNE, 15-fold lower at 48 h, p < 0.05) (Fig. 3a,e). Thus, although overexpression of Cygb in HSCs resulted in negligible protective effects on TAA-induced acute hepatocyte damage, the antioxidative defence system seemed to be functional in Cygb-TG mice.

### Inhibition of liver fibrosis in Cygb-TG mice under chronic TAA administration

Although overexpression of Cygb in HSCs had minor protective effects on TAA-induced acute liver damage, as shown in Fig. 3, HSCs^Cygb-TG^ were unlikely to become profibrogenic, as shown in Fig. 2. Accordingly, we hypothesized that overexpression of Cygb in HSCs might attenuate liver fibrosis in chronically insulted livers. Thus, we employed the TAA-induced chronic liver fibrosis model in both WT and Cygb-TG mice.

**Figure 4:**
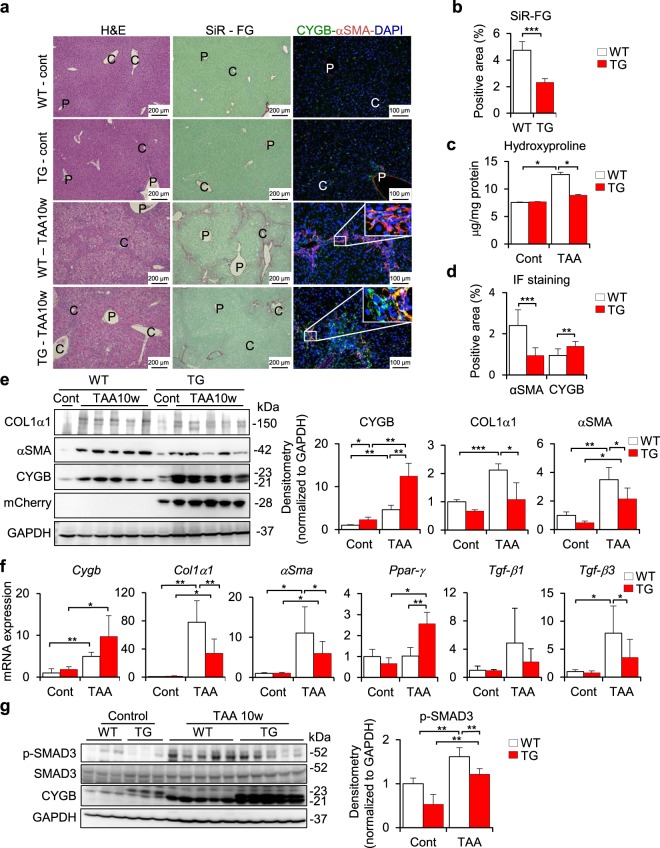
Inhibition of liver fibrosis development in long-term TAA-treated Cygb-TG mice. WT and Cygb-TG (TG) mice were subjected to TAA treatment for 10 weeks (TAA-10w). (**a**) Representative liver images of H&E, Sirius Red and Fast Green (SiR-FG) staining for collagen deposition and IF staining for the detection of αSMA (red) and CYGB (green); inset, 6x from the original pictures. P, portal vein; C, central vein. (**b**) Percentages of positive SiR-FG staining area per total areas of whole lobe liver sections were quantified (n = 5). (**c**) Hydroxyproline content in the liver tissue (µg/mg total protein) (n = 9 to 10). (**d**) Percentages of CYGB- and αSMA- positive areas per total areas of whole- lobe liver sections were quantified. (**e**) Immunoblot analysis shows COL1α1, αSMA, CYGB, and mCherry protein expression in the liver tissues of WT and TG mice in images and according to densitometric intensity quantification. GAPDH was used as a loading control and for normalization. Full-length Western blots of one gel are presented in Supplementary Fig. S8. (**f**) mRNA expression levels of *Cygb*, *mCherry*, *Col1α1*, *αSma*, *Tgf-β1* & *Tgf-β3*, and *Ppar-γ* in the liver were determined by RT-qPCR (*n* = 9 to 10). Levels were normalized to *Gapdh*. (**g**) Liver tissues from WT and TG mice were examined by immunoblotting for phosphorylated- and total SMAD3. The densitometric intensity of phosphorylated-SMAD3 was quantified. GAPDH was used as a loading control and for normalization. Full-length Western blots of one gel are presented in Supplementary Fig. S9. Data are expressed as the mean ± SD (n = 5). Values are given as the mean ± SD of all experiments. *p ≤ 0.05, **p ≤ 0.01, ***p ≤ 0.001.

After chronic liver injury induced by TAA treatment for 10 weeks, serum levels of AST and ALT showed no differences between Cygb-TG and WT mice (Supplementary Fig. S5a). However, while typical bridging fibrosis between the portal and central veins was observed in the livers of WT mice, it was markedly inhibited in Cygb-TG mice, as indicated by H&E staining as well as Sirius Red and Fast Green staining (SiR-FG) (Fig. 4a and Supplementary Fig. S5b). Quantification of collagen content in the liver was assessed by imaging determination of Sirius Red-positive areas and hydroxyproline content in the liver. Both of these techniques showed a significant reduction in the collagen content of Cygb-TG mouse livers relative to WT livers; the Sirius Red-positive area was 2-fold smaller (p < 0.0001) (Fig. 4b) and hydroxyproline level was reduced by 1.43-fold (p < 0.05) (Fig. 4c). Double immunofluorescence (IF) staining of CYGB (green) and αSMA (red) in TAA-treated WT livers revealed that almost all α-SMA-positive cells in the fibrotic lesion were also positive for CYGB (Fig. 4a, inset), indicating that they became activated. However, in TAA-treated Cygb-TG livers, some of the HSCs were positive for CYGB and negative for αSMA, indicating that they remained quiescent even in the fibrotic lesion (Fig. 4a, inset). When CYGB- and αSMA-positive areas were quantified, Cygb-TG livers showed decreased αSMA-positive areas (2.6-fold lower, p < 0.001), but CYGB-positive areas were 1.5-fold larger than those of the WT mice (p < 0.01) (Fig. 4d). Immunoblot and quantitative real-time polymerase chain reaction (RT-PCR) analyses clearly demonstrated significant downregulation of αSMA and COL1α1 and upregulation of CYGB in Cygb-TG livers (Fig. 4e,f). Interestingly, peroxisome proliferator-activated receptor-gamma (*Ppar-γ*), a marker of quiescent HSCs, was transcriptionally upregulated in Cygb-TG livers (Fig. 4f). Given that TGF-β is a major profibrogenic cytokine^28^, we examined the mRNA levels of *Tgf-β1* and 3 in TAA-treated livers and found that *Tgf-β3* was significantly downregulated in Cygb-TG livers relative to WT livers (Fig. 4f). Furthermore, mothers against decapentaplegic homolog 3 (SMAD3), an intracellular signalling molecule of the TGF-β pathway, showed decreased phosphorylation (normalized to glyceraldehyde 3-phosphate dehydrogenase, GAPDH) in Cygb-TG livers (Fig. 4g). Taken together, these results demonstrated that overexpression of Cygb in HSCs attenuates liver fibrosis development by reducing extracellular matrix production and blocking HSC activation regardless of TAA-induced hepatocyte damage.

### Liver inflammation was suppressed in Cygb-TG mice under chronic TAA administration

**Figure 5:**
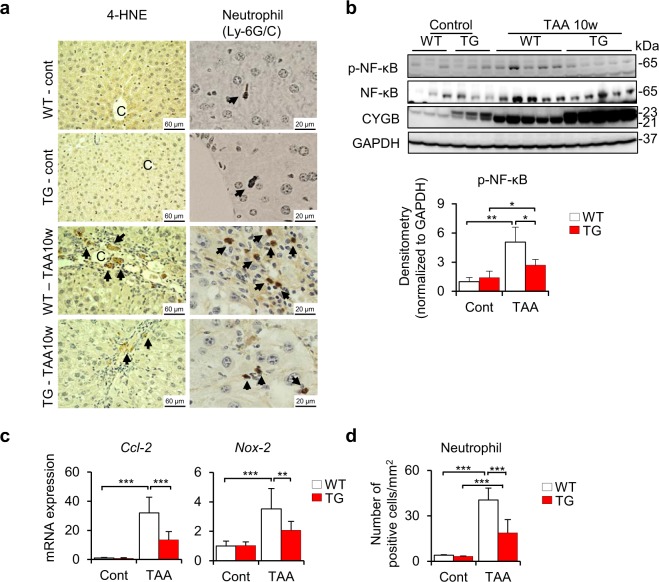
Cygb-TG mice exhibited ameliorated inflammation after 10 weeks of TAA treatment. WT and Cygb-TG (TG) mice were subjected to TAA treatment for 10 weeks (TAA-10w). (**a**) Representative images of liver sections were immunostained for 4-HNE and neutrophils. P, portal vein; C, central vein. (**b**) Liver tissues from WT and Cygb-TG mice were examined for immunoblotting for phosphorylated- and total NF-κB. The densitometric intensity of phosphorylated-NF-κB was quantified. GAPDH was used as the loading control and for normalization. Full-length Western blots in one gel are presented in Supplementary Fig. S10. (**c**) Hepatic mRNA levels of the attractant chemokines *Ccl-2* and *Nox-2* were determined by RT-qPCR (n = 9 to 10). (**d**) The average number of neutrophil-positive cells per square millimetre in liver sections from WT and TG mice was quantified (n = 5). Data are expressed as the mean ± SD. *p < 0.05, **p < 0.01, ***p < 0.001.

According to observations of acute TAA-induced liver injury (Fig. 3), which revealed the upregulation of *Nrf-2* and *Gpx-2* and the reduction of 4-HNE and 8-OHdG expression, ROS levels were assessed at the chronic phase. As expected, ROS were negligibly abundant in the chronic phase of TAA treatment as determined by immunohistochemical staining of 4-HNE, in which the positive area was limited around the fibrotic septa (Fig. 5a, left panels). The 4-HNE-positive area was barely detected in TAA-treated Cygb-TG livers (Fig. 5a, left panels). There was no significant difference in *Nrf-2* at both the mRNA and protein levels at this time -point between two groups (Supplementary Fig. S5c,d).

However, it was reported that TAA administration was associated with toxic hepatitis, which is primarily mediated by activation of the transcription factor nuclear factor (NF)-κB^29^. As shown in Fig. 5b, phosphorylated NF-κB (normalized to GAPDH) was induced in the TAA group (5-fold higher, p < 0.001) relative to control group, but considerably alleviated in TAA-treated Cygb-TG livers (1.51-fold lower, p < 0.05). These effects were concordant with the expression of NF-κB target genes, *Ccl-2* (2.4-fold lower, p < 0.001) and NADPH oxidase (*Nox-2*) (1.7-fold lower, p < 0.01) (Fig. 5c). Immunohistochemistry further confirmed the suppression of neutrophil accumulation in TAA-treated Cygb-TG livers relative to WT livers (2-fold lower, p < 0.0001) (Fig. 5a, right panels & d). Thus, overexpression of Cygb in HSCs attenuated inflammatory reactions in addition to fibrogenesis under chronic TAA treatment.

### Alleviation of ROS-induced activation of HSCs by overexpression of Cygb

**Figure 6:**
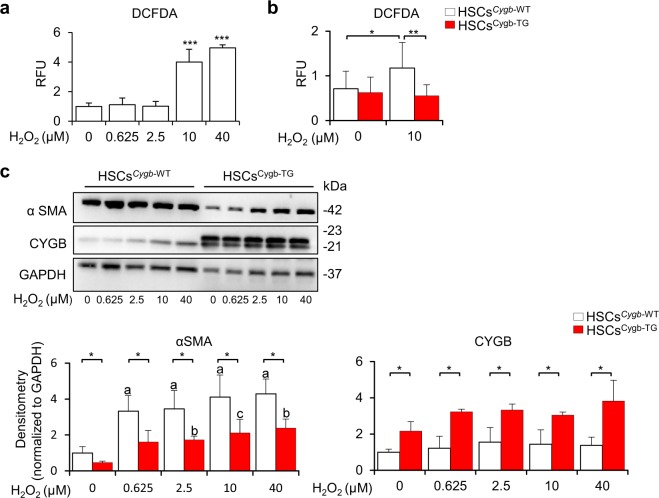
Overexpression of Cygb alleviated the ROS-induced activation of HSCs. DCFDA assay for HSCs^*Cygb-*WT^ treated with H_2_O_2_ in a dose-dependent manner (**a**), and comparison to HSCs^Cygb-TG^ at a specific concentration (10 μM) (**b)**. RFU, relative fluorescence unit. (**c**) Immunoblot analysis of αSMA and CYGB in HSCs^*Cygb-*WT^ and HSCs^Cygb-TG^ under H_2_O_2_ exposure at different doses. Densitometric intensity was quantified. GAPDH was used as a loading control and for normalization. a, p ≤ 0.001 relative to non-treated HSCs^*Cygb-*WT^; b, p ≤ 0.01 relative to non-treated HSCs^*Cygb-*TG^; c, p ≤ 0.05 relative to non-treated HSCs^*Cygb-*TG^. Full-length Western blots of one gel are presented in Supplementary Fig. S11. Values are given as the mean ± SD of all experiments. *p < 0.05, **p < 0.01, ***p < 0.001.

Because CYGB is known to scavenge reactive oxygen species (ROS)^30–34^, we hypothesized that Cygb overexpression may inhibit HSC activation and fibrosis development by protecting HSCs from ROS stress. To test this hypothesis, primary mouse HSCs were isolated from both WT and Cygb-TG livers and cultured on plastic dishes for *in vitro* activation. On day 2 of cell culture, they were exposed to hydrogen peroxide (H_2_O_2_) at different concentrations and harvested at day 4 to examine αSMA protein expression. ROS formation, as indicated by DCFDA assays, was dose-dependently induced in HSCs^*Cygb*-WT^ under H_2_O_2_ treatment (Fig. 6a) but attenuated in HSCs^Cygb-TG^ with 10 μM of H_2_O_2_ (Fig. 6b). αSMA expression was significantly suppressed in H_2_O_2_-treated HSCs^Cygb-TG^ relative to HSCs^*Cygb*-WT^ (Fig. 6c). Thus, these results revealed that overexpression of Cygb inhibited ROS-induced αSMA expression in HSCs.

## Discussion

### Generation and application of Cygb transgenic mice

The function of CYGB has been debated since its first discovery just over a decade ago by our group^3^ and Burmester *et al*.^1^. We have established *Cygb*-deficient mice and published several studies showing their high sensitivity to fibrosis and cancer development despite the different aetiologies of these conditions^20–22,35^. Then, we asked whether overexpression of Cygb can rescue these manifestations. Thus, we generated a new mouse strain, Cygb-TG, which stably expressed 10 copies of the Cygb gene since approximately 10 copies were reported to be ideal for analysis^36^. Exogenous *Cygb* was incorporated with a *2A-mCherry* tag under the regulation of the *Cygb* promoter, which directs gene expression in target *Cygb*-expressing cells. The mCherry reporter, which expresses a monomeric red fluorescent protein, is favourable for live- imaging studies without cellular toxicity^37^. These mice were used for *Cygb* gene/protein expression profiling under physiological or pathological conditions. Furthermore, these transgenic mice are novel resources for the purification of Cygb-expressing cells, such as HSCs, from various other cells in the liver. It will be important to track mCherry reporter-tagged cells to observe the lineage specification and differentiation of Cygb-expressing cells and their origin using these mice in the near future.

CYGB is ubiquitously expressed in multiple tissues^1,6,9^. Notably, CYGB expression is high in lipid-containing tissues, such as fat tissue or the brain, and in the liver, it was specifically expressed in quiescent HSCs containing lipid droplets for vitamin A storage (Figs 1e,f and 2). Exogenous CYGB expression in transgene mice was also highest in fat-related tissues (Fig. 1e,f). It was suggested that CYGB is involved in lipid metabolism^38,39^. Of note, only quiescent HSCs store vitamin A, and therefore, it is likely that CYGB is necessary for the quiescent status of HSCs. This finding suggests that CYGB could be used as a quiescent marker of HSCs. The preferential expression of CYGB suggests that there is an important functional benefit or role uniquely provided by CYGB in these tissues, which should be further analysed.

### ROS in fibrosis and the role of Cygb

Almost 50 years have passed since the first report describing the role of ROS in liver injury induced by CCl_4_ through lipid peroxidation by Comporti *et al*.^40^ and Ghoshal *et al*.^41^ in 1965, and oxidative stress is common to various types of chronic liver injury and hepatic fibrosis, not only in ethanol-induced^42,43^, CCl_4_-induced^44^, and non-alcoholic fatty liver diseases^45^ but also in conditions induced by iron overload^46,47^ and hepatitis C virus^48^. In these pathological conditions, HSC activation is triggered by ROS and plays a critical role in extracellular matrix remodelling and fibrosis progression^16,49^. Remarkably, CYGB was initially found in rat HSCs with increased expression under activated conditions^3^; thus, it was hypothesized that CYGB expression might protect HSCs from exposure to endogenous and exogenous ROS during liver injury. The ROS scavenger function of CYGB is evidenced by its ability to detoxify radicals via reaction with its haem^18,50^. We previously showed that genetic knockout of *Cygb* promotes hepatic fibrosis and the development of hepatocellular carcinoma, accompanied by increased markers of oxidative stress under the administration of diethylnitrosamine^20^ or a choline-deficient L-amino acid defined diet^21^, and that both primary HSCs^*Cygb-*null^ and *siCygb*-treated HSCs^*Cygb-*WT^ exhibited increased ROS generation and upregulated expression of collagen alpha1(I), *Tim-1*, *Il-6*, and *Tnfα*^21^. In humans, decreased expression of *CYGB* was also found in patients with advanced fibrosis due to HCV infection^51^, NASH, and HCC^21^. In contrast, it was reported that *CYGB* overexpression *in vitro* rescued the human neuronal cell line TE671 from pro-oxidant Ro19-8022-induced DNA damage^32^ and protected human neuroblastoma SH-SY5Y cells from H_2_O_2_-induced cell death^33,34^. In this study, chronic TAA administration in Cygb-overexpressing mice produced consistent results with clear inhibition of oxidative stress, inflammation and fibrosis relative to WT mice (Figs 3–5). Interestingly, when we isolated primary HSCs from both WT and Cygb-TG mice and challenged them with different doses of H_2_O_2_ to induce oxidative stress, we found that H_2_O_2_ dose-dependently induced αSMA expression in HSCs^*Cygb-*WT^, while this effect of H_2_O_2_ was attenuated, at least in part, in HSCs^Cygb-TG^. Thus, induction and maintenance of CYGB in HSCs is relevant to the development of promising antifibrotic drug therapies.

### HSC activation and the role of Cygb

Liver injury triggers HSC activation, which has been identified as a key event in hepatic fibrogenesis. The activation process is complex, but one of its most prominent features is the synthesis of high levels of extracellular matrix materials, resulting in the deposition of scar or fibrous tissue^52^. Thus, a number of studies have been performed to maintain HSC quiescence, deactivate HSCs or remove activated HSCs by inducing apoptosis of these cells to regress liver fibrosis^53–56^. Regulation of the transcriptional activity of PPAR-γ, a well-known marker of quiescent HSCs, can modulate HSC activation^57^. Here, we found that the transcriptional level of *Ppar-γ* was significantly increased in Cygb-TG livers relative to WT livers (Fig. 4f) and, conversely, decreased in *CYGB-*silenced human hepatic stellate cells (HHSteC) (unpublished data). Although we have not uncovered the mechanism underlying how CYGB regulates *Ppar-γ* mRNA expression, we speculate that *Ppar-γ* expression is a characteristic of quiescent HSCs that is maintained by CYGB during TAA-induced fibrosis. In contrast to *Ppar-γ*, increased NF-κB activity was reported to be induced during HSC activation and promote HSC survival, contributing to fibrotic progression^58,59^. Of note, NF-κB phosphorylation was increased 5-fold in TAA-treated WT mice but significantly inhibited in Cygb-TG livers (Fig. 5b). Thus, overexpression of Cygb was demonstrated to be relevant to the factors involved in the maintenance of HSC quiescence, such as PPAR-γ, and to the effective reduction of NF-κB activity, both of which inhibit HSC activation, resulting in decreased fibrosis.

### Limitations and future works

Although we observed marked attenuation of fibrosis development in Cygb-TG mice relative to WT mice under chronic TAA treatment, the hepatocyte damage indicated by serum AST and ALT levels was only slightly changed. These findings indicate that (1) overexpression of Cygb failed to protect hepatocytes against TAA-induced toxicity; (2) TAA at a single dose of 50 mg was toxic to the mice, which eclipsed the protective role of CYGB; and (3) even though hepatocyte damage occurred in the acute phase in both WT and Cygb-TG mice at the same magnitude, the chronic phase of TAA treatment, characterized by fibrosis development, was attenuated in Cygb-TG mice, which may reflect the major role of HSCs in this situation. We observed that Cygb overexpression induced the transcriptional downregulation of *Tgf-β3*, the suppression of SMAD3 phosphorylation and the inhibition of COL1α1 and αSMA at both the protein and RNA levels (Fig. 4). However, for the next step, we need to verify whether overexpression of Cygb can directly suppress *Tgf-β* expression, which is very important when considering future antifibrosis therapy.

In conclusion, our data revealed that overexpression of Cygb in HSCs attenuates HSC activation and fibrosis development under chronic TAA administration. Based on these data together with our previous studies using *Cygb*-deficient mice, we hypothesize that CYGB is a potent regulator of HSC activation and is involved in all aspects of hepatic inflammation, fibrosis and cancer development. Continued elucidation of CYGB function is crucial, particularly the complex cross-talk between CYGB-positive HSCs and epithelial cells (hepatocytes and bile-duct cells) and inflammatory cell subsets.

## Materials and Methods

### BAC Transgenic Construct

A mouse Cygb BAC clone, RP23-330N7, was selected from the RPCI-23 Female C57BL/6 J Mouse BAC Library by a search of the mouse BAC end database at the National Center for Biotechnology Information (NCBI). The BAC end sequences indicate that the BAC clone contains the entire 8.7- kb mouse Cygb genomic sequence, with an additional 80 and 123 kb of 5′- and 3′-flanking genomic DNA around the mouse Cygb gene, respectively (Fig. 1a). The BAC clone was obtained from the BACPAC Resources Center at Children’s Hospital Oakland Research Institute (CHORI).

A chimeric Cygb-2A-mCherry BAC transgenic construct harbouring the 2A-mCherry reporter gene in place of the stop codon of the Cygb gene locus was generated by BAC recombineering. The 2A-mCherry reporter gene was transferred to the Cygb BAC clone by a Red/ET Counter Selection BAC Modification Kit (Gene Bridges, Heidelberg, Germany)^60^. In brief, an rpsL-neo counter selection cassette flanked by two adjacent sequences of the stop codon of the Cygb gene was amplified by PCR. The amplified rpsL-neo counter selection cassette was inserted in the Cygb gene of the BAC clone by Red/ET recombination. The 2A-mCherry reporter gene, flanked by 110 bp of the upstream sequence of Cygb gene stop codon and 87 bp of the downstream sequence of its stop codon, was synthesized (Fasmac, Kanagawa, Japan). The modified 2A-mCherry gene fragment was precisely transferred to the Cygb gene of the mouse BAC clone by Red/ET recombination to construct a chimeric Cygb-2A-mCherry BAC clone. BAC modification was verified by sequencing.

The Cygb-2A-mCherry BAC transgenic construct was purified for microinjection with slight modification of the procedure described by Abe^61^. The BAC transgenic construct was extracted from 250 ml of *E. coli* culture using a Nucleobond Plasmid Purification Kit (Macherey-nagel, Duren, Germany). For purification, 10 μg of the BAC transgenic construct was linearized overnight with PI-SceI endonuclease (New England Biolabs) to cleave the unique site in the BACe3.6 vector sequence. The linearized BAC DNA was separated by pulsed field gel electrophoresis (PFGE) and extracted from the preparative pulsed field gel by electroelution. After dialysis against TE buffer containing 0.1 mM EDTA, aliquots were subjected to PFGE for size analysis and quality control. The BAC DNA concentration was adjusted to 1 ng/μl for microinjection. Aliquots of BAC DNA solution were stored at 4 °C until microinjection.

### Cygb-2A-mCherry BAC Transgenic Mice

Cygb-2A-mCherry BAC transgenic mice were generated by pronuclear injection of C57BL/6JJcl embryos (Clea Japan Inc., Kanagawa, Japan). Transgenic founders of the BAC transgenic construct were assessed by Southern blotting of XbaI-digested tail DNA probed by the [^32^P]-labelled mCherry gene fragment. Nine of 65 progenies (14%) contained the transgene, as detected by Southern blotting of tail DNA. Several transgenic founder mice were bred with mice of the same strain. Only mice with 10 copies of the Cygb-transgene were used for this study. No obvious gross phenotypic differences were apparent in a comparison of transgene-positive and transgene-negative littermates at birth and until 24 months old.

All mice received humane care according to Guide for the Care and Use of Laboratory Animals, National Institutes of Health. All protocols and experimental procedures were approved by the Institutional Animal Care and Use Committee of Osaka City University and performed in accordance with the guidelines of the National Institutes of Health for the use of animals in research. Mice were housed in a temperature-controlled (24 ± 1 °C) environment, with humidity levels of 55 ± 5% and alternating 12-h light/12-h dark cycles. They had free access to water and standard rodent diet.

### Genotyping of Cygb Transgenic Mice

The founder Cygb-TG mice were backcrossed with C57BL/6 J wild type mice for 3–6 generations, and only their offspring with 10 Cygb copies were used in this study. The litter sizes were normal, and all generated mice were genotyped by real-time PCR at the age of 6 weeks. The relative copy numbers of both Cygb and mCherry DNA were quantified by using tail biopsy specimens. DNA isolated from the tail biopsies of Cygb 10 copies-transgenic founder (TgFD10c) mice was used as a positive control, and DNA from 1 copy founder (TgFD1c) mice was used as a reference sample. The primer pair used for Cygb was FW-5′cgcctccatcttggccattc-3′ and RV-5′-agggcgagcacagaggatac-3′ and for mCherry was FW-5′cccgccgacatccccgacta-3′ and RV-5′- gggtcacggtcaccacgcc-3′. *Gapdh* levels were used to normalize the relative DNA levels. The analysis of 747 offspring revealed the presence of 10 copies in Cygb-TG mice at a frequency of 43%.

### Analysis of the phenotype of Cygb transgenic mice

Cygb-TG and WT mice were observed until they were 24 months old. All tissues of Cygb-TG and WT mice, including the brain, heart, lung, liver, kidney, spleen, pancreas, fat, intestine, colon, skin, testis, and uterus, were collected and live imaged using an IVIS Imaging system (Caliper Life Sciences, Inc., Hopkinton, MA, USA). Tissues were isolated to examine CYGB and mCherry protein and mRNA levels in Cygb-TG mice relative to WT mice.

### Blood pressure (BP) measurement

BP was measured in conscious wild type (n = 51) and Cygb-TG (n = 35) mice using the tail-cuff method (CODA-2, Kent Scientific, Torrington, CT). Briefly, a cuff was placed on the tail of the mice to occlude the blood flow. Upon deflation, the blood pressure sensors, which were placed distal to the occlusion cuff, monitored the blood pressure via volume pressure recording (VPR). VPR used a specially designed differential pressure transducer to noninvasively measure the blood volume in the tail. Data from each mouse were obtained from one accepted session, which consisted of 5 acclimatization cycles followed by 15 BP measurement cycles; a set was accepted if the computer identified >50% successful readings. The average value from the accepted session was used for systolic BP (SBP) and diastolic BP (DBP) in each individual mouse.

### TAA Treatment

#### Acute model

Acute liver damage was induced by intraperitoneal (*i.p*.) injection of a single dose of thioacetamide (TAA) at 50 mg/kg body weight (BW) to 12-week-old Cygb-TG and WT mice at 5 mice per group. Mice were sacrificed after 24 or 48 h of TAA (Sigma, St Louis, MO) treatment, and samples were obtained. Healthy controls were given only an adequate saline solution by *i.p*. injection.

#### Chronic model

Cygb-TG and wild type mice, 10 mice per group, were given an *i.p*. injection of an escalating dose of TAA twice a week^62^. For the first week, mice received an *i.p*. injection of 50 mg/kg TAA for the first dose and then 100 mg/kg TAA for the second dose; for weeks 2–3, they received 200 mg/kg twice a week; for weeks 4–5, they received 300 mg/kg twice a week; and for weeks 6–10, they received 400 mg/kg twice a week. Mice were sacrificed 2 days after the last TAA application. Healthy controls were given only an adequate saline solution by *i.p*. injection.

### Necropsy

At necropsy, mice were weighed, anaesthetized, and examined for grossly visible lesions in whole organs. All tissues were collected, weighed (in the case of liver), and examined for macroscopic lesions. For histological examination, 2 to 3 mm-thick sections from tissues were fixed in 10% formalin for 24 h and embedded in paraffin. The samples were then sectioned at 5 µm, and H&E, Sirius Red Fast Green, and immunohistochemistry staining were performed^21^. For RNA, protein and biochemistry examinations, 20–30 mg of tissue was stored at −80 °C until analysis.

### Histological, Immunohistochemistry, and Immunofluorescence Analysis

H&E staining, immunohistochemistry and immunofluorescence analysis were performed as described previously^20^. The primary antibodies used are listed in Supplementary Table S3. Polyclonal antibodies against CYGB were generated in our laboratory^3,20,51^. For quantification of liver fibrosis, 5-µm-thick sections were stained with PicroSirius Red (Sigma-Aldrich, Tokyo, Japan) and counterstained with Fast Green dye (Sigma-Aldrich) (SiR-FG). Each section was imaged separately at 100 times magnification by a BZ-X700 microscope (Keyence, Osaka, Japan) and merged into whole- lobe pictures by using BZ-X Analyser software. Percentages of SiR-FG- positive areas per corresponding lobe area were calculated.

### Immunoblotting

Proteins isolated from tissues (30 µg) or from HSCs (3.5–7 µg) were subjected to SDS-PAGE and transferred to Immuno-Blot® PVDF membranes (Bio-Rad, California, USA). After the membranes were blocked by 5% skim milk, they were probed with the following primary antibodies: anti-CYGB (1:2000; our laboratory), anti-mCherry (1:1000; Abcam, Japan), anti-αSMA (1:2000; DAKO, UK), anti-phosphorylated- and total-SMAD3 (1:1000; Abcam, Japan), anti-phosphorylated- and total-NF-κB (1:1000; Cell Signaling, Japan), or anti-GAPDH (1:2000; Santa Cruz Biotechnology, Santa Cruz, CA). Membranes were then labelled with horseradish peroxidase–conjugated secondary antibodies (1:2000). Immunoreactive bands were visualized by enhanced chaemiluminescence using L-012 substrate (ImmunoStar LD, Wako, Osaka, Japan) and documented with a Fujifilm Image Reader LAS-3000 (Fujifilm, Tokyo, Japan) coupled to image analysis software (Multi Gauge, Fujifilm).

### Quantitative Real-Time PCR

Total RNA was extracted from cells, liver and other tissues using the RNeasy Mini Kit (Qiagen, Valencia, CA). Then, 1 µg or 200 ng of total RNA extracted from tissues or cells, respectively, was used for the synthesis of cDNA by oligo (dT)_12–18_ primers (ReverTra Ace, Toyobo, Osaka, Japan) according to the manufacturer’s instruction. Gene expression was measured by quantitative real-time PCR using cDNA, THUNDERBIRD SYBR qPCR Mix Reagents (Toyobo), and a set of gene-specific oligonucleotide primers and probes (Supplementary Table S4) using an Applied Biosystems Prism 7500 system (Applied Biosystems, Tokyo, Japan). *Gapdh* levels were used to normalize relative mRNA abundance.

### AST and ALT Activities

Serum AST and ALT activities were analysed using a commercially available kit (Wako, Osaka, Japan) according to the manufacturer’s protocol. AST and ALT levels are expressed as international units per litre (IU/l).

### Hydroxyproline Assay

Hydroxyproline content in the liver was measured by a spectrophotometric assay with a Hydroxyproline Assay Kit (BioVision, Milpitas, CA) according to the assay protocol. Briefly, liver tissue was homogenized in ice-cold distilled water (100 µl of water for every 10 mg of tissue) using a bead cell disrupter (TOMY, Tokyo, Japan). Subsequently, one volume of 12 N HCl was added to each homogenized sample in a pressure-tight, Teflon-capped vial and hydrolysed for 3 h at 120 °C. After hydrolysis, 10 ml of each hydrolysed sample was transferred to a 96-well plate and evaporated to dryness under a vacuum. Then, samples were oxidized with chloramine-T for 5 min at room temperature. The reaction mixture was then incubated in dimethylamino-benzaldehyde at 60 °C for 90 min and cooled to room temperature. A series of wells containing hydroxyproline standards was prepared for each assay. Sample absorbance was measured at 560 nm. Hydroxyproline content is expressed as microgram of hydroxyproline per mg of total liver protein.

### Cell Culture

HSCs were isolated from non-treated wild- type (HSC^*Cygb-*WT^) and Cygb*-*TG mice (HSC^Cygb-TG^) by the pronase-collagenase digestion method as previously described^63^. Briefly, normal livers were perfused for 3 min with SC-1 solution consisting of 8,000 mg/L NaCl, 400 mg/L KCl, 88.17 mg/L NaH_2_PO_4_. 2H_2_O, 120.45 mg/L Na_2_HPO_4_, 2,380 mg/L HEPES, 350 mg/L NaHCO_3_, 190 mg/L EGTA, and 900 mg/L glucose, pH 7.3, followed by 10 min with 0.1% pronase E (Merck, Tokyo, Japan) and 10 min with 0.05% collagenase (Wako, Osaka, Japan) dissolved in SC-2 solution consisting of 8,000 mg/L NaCl, 400 mg/L KCl, 88.17 mg/L NaH_2_PO_4_.2H_2_O, 120.45 mg/L Na_2_HPO_4_, 2,380 mg/L HEPES, 350 mg/L NaHCO_3_, and 560 mg/L CaCl_2_.2H_2_O, pH 7.3. The digested livers were excised, cut into small pieces, and incubated at 37 °C in SC-2 solution containing 0.04% pronase E, 0.04% collagenase, and 20 g/ml of DNase I (Roche, Mannheim, Germany). The resulting suspension was filtered through a 70 µm nylon cell strainer (BD Falcon, CA, US) and centrifuged on an 8.2% Nycodenz (Axis-shield PoC AS, Norway) cushion, which produced a stellate cell–enriched fraction in the upper whitish layer. The cells were washed, suspended in Dulbecco’s modified Eagle’s medium (Gibco, MA, USA) supplemented with 10% foetal bovine serum (Gibco) and antibiotics (100 U/ml penicillin and 100 mg/ml streptomycin), plated on 24-well plastic culture dishes (Greiner Bio-One, Tokyo, Japan), and incubated at 37 °C in a 5% CO_2_ air environment. Cell purity was approximately 95% as assessed by the typical star-like shape with a lipid droplet configuration.

HSCs were plated overnight, and the culture medium was subsequently washed 2 times with PBS to remove dead cells and cell debris. Attached HSCs^*Cygb*-WT^ and HSCs^Cygb-TG^ were analysed for spontaneous RNA and protein expression at days 1, 4 and 7. For hydrogen peroxide (H_2_O_2_) treatment, primary mouse HSCs isolated from both WT and Cygb-TG livers were exposed to 0.625, 2.5, 10, and 40 µM H_2_O_2_ on day 2 and harvested at day 4 of cell culture to examine protein and RNA expression of fibrosis-related genes.

### DCFDA assay

The DCFDA-Cellular Reactive Oxygen Species Detection Assay using the cell-permeable reagent 2′,7′-dichlorofluorescin diacetate (DCFDA) (Abcam, Tokyo, Japan) to measure intracellular hydroxyl, peroxyl and other ROS activity was performed according to the assay protocol. Briefly, HSCs^*Cygb-*WT^ and HSCs^Cygb-TG^ were induced to produce ROS by different doses of H_2_O_2_ (0.625 to 40 µM) administrated on day 2 of cell culture. After 48 h of H_2_O_2_ treatment, both the stimulated and non-treated cells were washed twice with PBS and incubated with a working concentration of DCFDA (25 mM) for 30 min at 37 °C. After diffusion into the cell, DCFDA is deacetylated by cellular esterases to a non-fluorescent compound, which is later oxidized by ROS into 2′, 7′-dichlorofluorescein (DCF). DCF is a highly fluorescent compound that can be detected by fluorescence spectroscopy with maximum excitation and emission spectra of 495 nm and 529 nm, respectively.

### Data Analysis

The data presented as bar graphs are the means ± SDs in all experiments. Statistical analyses were performed using Student’s *t*-test, and p < 0.05 indicated statistical significance.

## Electronic supplementary material

Supplementary Information
