# Supplementary material for: Selective overexpression of cytoglobin in stellate cells attenuates thioacetamide-induced liver fibrosis in mice
Source: Sci Rep. 2018 Dec 14;8:17860. doi: 10.1038/s41598-018-36215-4 (PMC6294752; doi:10.1038/s41598-018-36215-4)
Supplement: Supplementary file 1 — Supplementary Information [file 41598_2018_36215_MOESM1_ESM.pdf]

## Supplementary Information

# Selective overexpression of cytoglobin in stellate cells attenuates thioacetamide-induced liver fibrosis in mice

Nguyen Thi Thanh Hai[1,2,*], Le Thi Thanh Thuy[1,*], Akira Shiota[3], Chiho Kadono[1], Atsuko Daikoku[1], Dinh Viet Hoang[1], Ninh Quoc Dat[1], Misako Sato-Matsubara[1], Katsutoshi Yoshizato[1,2,4] & Norifumi Kawada[1†]

From the [1]Departments of Hepatology, Graduate School of Medicine, Osaka City University, Osaka, Japan. [2]Department of Biochemistry, Hanoi Medical University, Hanoi, Vietnam. [3]PhoenixBio Co. Ltd., Hiroshima, Japan. [4]Endowed Laboratory of Synthetic Biology, Graduate School of Medicine, Osaka City University, Osaka, Japan.

[*]These authors contributed equally to this work.

[†]**Corresponding author:**

Department of Hepatology, Graduate School of Medicine, Osaka City University, 1-4-3 Asahimachi, Abeno, Osaka 545-8585, Japan

Phone: +81-6-6645-3897; Fax: +81-6-6646-6072;

E-mail: kawadanori@med.osaka-cu.ac.jp

**Supplementary Table S1**: Biochemistry Blood Tests of WT and *Cygb*-TG Mice

| Parameter | WT (n=3, mean ± SD) | TG (n=5, mean ± SD) | *p* value |
|---|---|---|---|
| ALB (g/dL) | 3 ± 0.12 | 3 ± 0.18 | Not significant |
| AST (IU/L) | 37 ± 1.00 | 39 ± 6.76 | Not significant |
| ALT (IU/L) | 23 ± 4.16 | 18 ± 1.50 | Not significant |
| LDH (IU/L) | 158 ± 45.98 | 193 ± 69.64 | Not significant |
| LAP (IU/L) | 15 ± 1.15 | 16 ± 3.88 | Not significant |
| ChE (IU/L) | 22 ± 1.53 | 25 ± 6.76 | Not significant |
| T-CHO (mg/dL) | 84 ± 7.51 | 75 ± 9.29 | Not significant |
| F-CHO (mg/dL) | 23 ± 3.06 | 22 ± 4.63 | Not significant |
| E-CHO (mg/dL) | 60 ± 4.73 | 53 ± 4.90 | Not significant |
| TG (mg/dL) | 82 ± 14.01 | 93 ± 59.71 | Not significant |
| LDL-C (mg/dL) | 4 ± 2.00 | 5.6 ± 1.50 | Not significant |
| HDL-C (mg/dL) | 52 ± 4.73 | 41 ± 5.37 | $p < 0.05$ |
| T-BIL (mg/dL) | 0.05 ± 0.01 | 0.064 ± 0.02 | Not significant |
| D-BIL (mg/dL) | 0.006 ± 0.01 | 0.01 ± 0.01 | Not significant |
| I-BIL (mg/dL) | 0.04 ± 0.02 | 0.054 ± 0.02 | Not significant |
| TBA (μmol/L) | 2.67 ± 1.15 | 3.75 ± 1.48 | Not significant |

**Supplementary Table S2**: Hematology Tests of WT and *Cygb*-TG Mice

| Parameter | WT (n=7, mean ± SD) | TG (n=8, mean ± SD) | *p* value |
|---|---|---|---|
| WBC (/μL) | 5811 ± 1452 | 4433 ± 2102 | Not significant |
| RBC (×104/μL) | 791 ± 138 | 792 ± 112 | Not significant |
| Hb (g/dL) | 13 ± 2 | 13 ± 1 | Not significant |
| Ht (%) | 46 ± 6 | 45 ± 5 | Not significant |
| MCV (fL) | 58 ± 4 | 57 ± 3 | Not significant |
| MCH (pg) | 16 ± 1 | 16 ± 1 | Not significant |
| MCHC (%) | 27 ± 1 | 28 ± 1 | Not significant |
| PLT (×104/μL) | 104 ± 29 | 92 ± 15 | Not significant |

**Supplementary Table S3.** Summary of Primary Antibodies Used for Immunohistochemistry or Immunofluorescences

| **Antigen*** | **Source** | **Name/clone†; catalog no.** | **Incubation‡** |
|---|---|---|---|
| αSMA | Dako | Monoclonal (Mo); M0851 | O/N 4ºC, 1:100 |
| COL1α1 | Rockland | Polyclonal (Rb); 600-401-103-0.1 | O/N 4ºC, 1:100 |
| CYGB | Our laboratory | Polyclonal (Rb); | O/N 4ºC, 1:100 |
| CD31 | R&D System | Polyclone (Go); AF3628 | O/N 4ºC, 1:100 |
| Desmin | Invitrogen | Polyclone (Rb); PA5-16705 | O/N 4ºC, 1:100 |
| mCherry | Abcam | Monoclonal (Mo); ab125096 | O/N 4ºC, 1:100 |
| 4-HNE | Abcam | Polyclonal (Rb); ab46545 | O/N 4ºC, 1:50 |
| 8-OHdG | Abcam | Polyclonal (Go); ab10802 | O/N 4ºC, 1:100 |
| Neutrophil (Ly-6G and Ly-6C) | Abcam | Monoclonal (Rt); ab2557 | O/N 4ºC, 1:100 |
| NRF-2 | Abcam | Polyclonal (Rb); ab137550 | O/N 4ºC, 1:100 |

*All antigens were retrieved by autoclaving for 15 min in 0.01 mol/L citrate buffer containing 0.05% Tween 20 (pH 6.0), except for Neutrophil, in which proteinase K (400 mg/mL) in TE buffer (pH 8.0) was used for 5 min incubation at room temperature.

†Rb, rabbit; Mo, mouse; Rt, rat; Go, goat

‡ O/N, overnight

**Supplementary Table S4:** Mouse Primers Used for Quantitative Real-Time PCR

| **Gene** | **Sequence** |
|---|---|
| *αSma* | *Forward* TCCCTGGAGAAGAGCTACGAACT<br>*Reverse* AAGCGTTCGTTTCCAATGGT |
| *Col1α1* | *Forward* GAGCGGAGAGTACTGGATCG<br>*Reverse* GTTCGGGCTGATGTACCAGT |
| *Cygb* | *Forward* CCTGGTGAGGTTCTTTGTGAAC<br>*Reverse* CCAAGGGATCCTCCATGTGT |
| *Gapdh* | *Forward* TGCACCACCAACTGCTTAG<br>*Reverse* GGATGCAGGGATGATGTTC |
| *Gpx-2* | *Forward* GGCGTCACTCTGAGGAACAAC<br>*Reverse* GCGACATTGCAGCTCATTGA |
| *mCherry* | *Forward* CCCGCCGACATCCCCGACTA<br>*Reverse* GGGTCACGGTCACCACGCC |
| *Ccl-2* | *Forward* GAGAGCCAGACGGGAGGAAG<br>*Reverse* TGAATGAGTAGCAGCAGGTGAG |
| *Nox-2* | *Forward* CCCTTTGGTACAGCCAGTGAAGAT<br>*Reverse* CAATCCCGGCTCCCACTAACATCA |
| *Nrf-2* | *Forward* CGAGATATACGCAGGAGAGGTAAGA<br>*Reverse* GCTCGACAATGTTCTCCAGCTT |
| *Ppar-γ* | *Forward* GATGCACTGCCTATGAGCAC<br>*Reverse* TCTTCCATCACGGAGAGGTC |
| *Tgf-β1* | *Forward* GGAGAGCCCTGGATACCAAC<br>*Reverse* AAGTTGGCATGGTAGCCCTT |
| *Tgf-β3* | *Forward* AGGGCCCTGGACACCAATTAC<br>*Reverse* CCTTAGGTTCTGGGACCCATTTC |

# Supplementary Figure S1

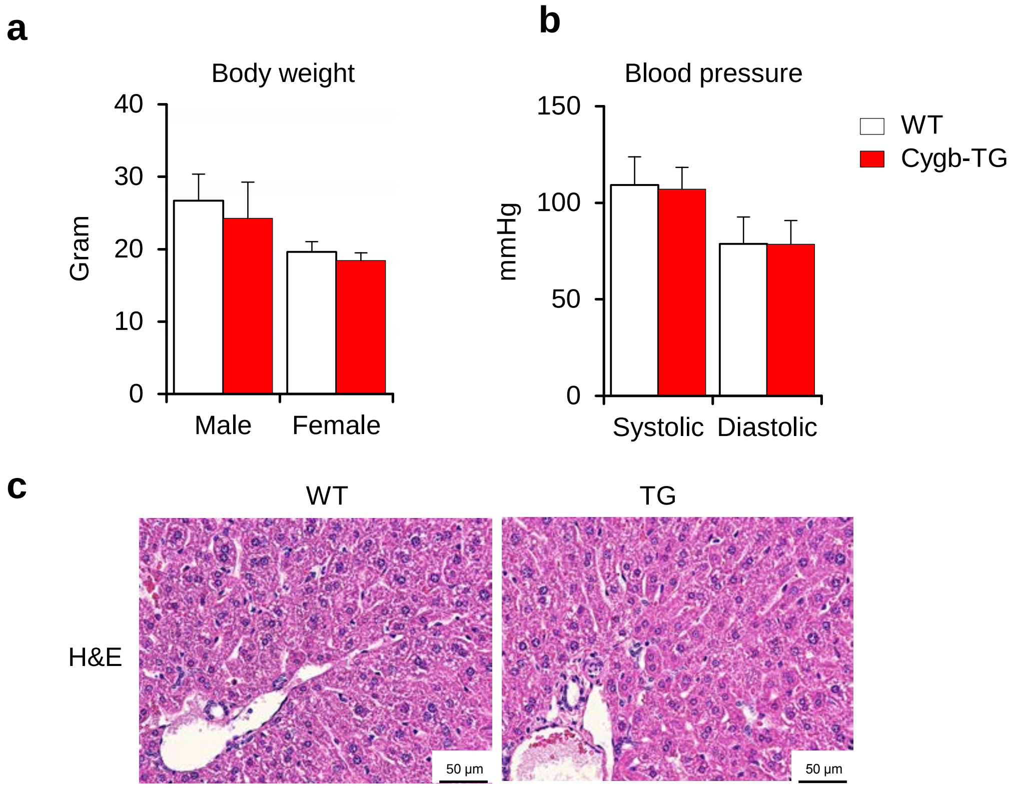


**Supplementary Figure S1. a**. Body weight of Cygb-TG mice and their WT littermates (n=8-12). **b**. Blood pressure of Cygb-TG mice (n= 35) and WT mice (n=51). Data are expressed as the mean ± SD. **c**. H&E staining of liver from wild -type (WT) and Cygb-TG mice (TG).

# Supplementary Figure S2

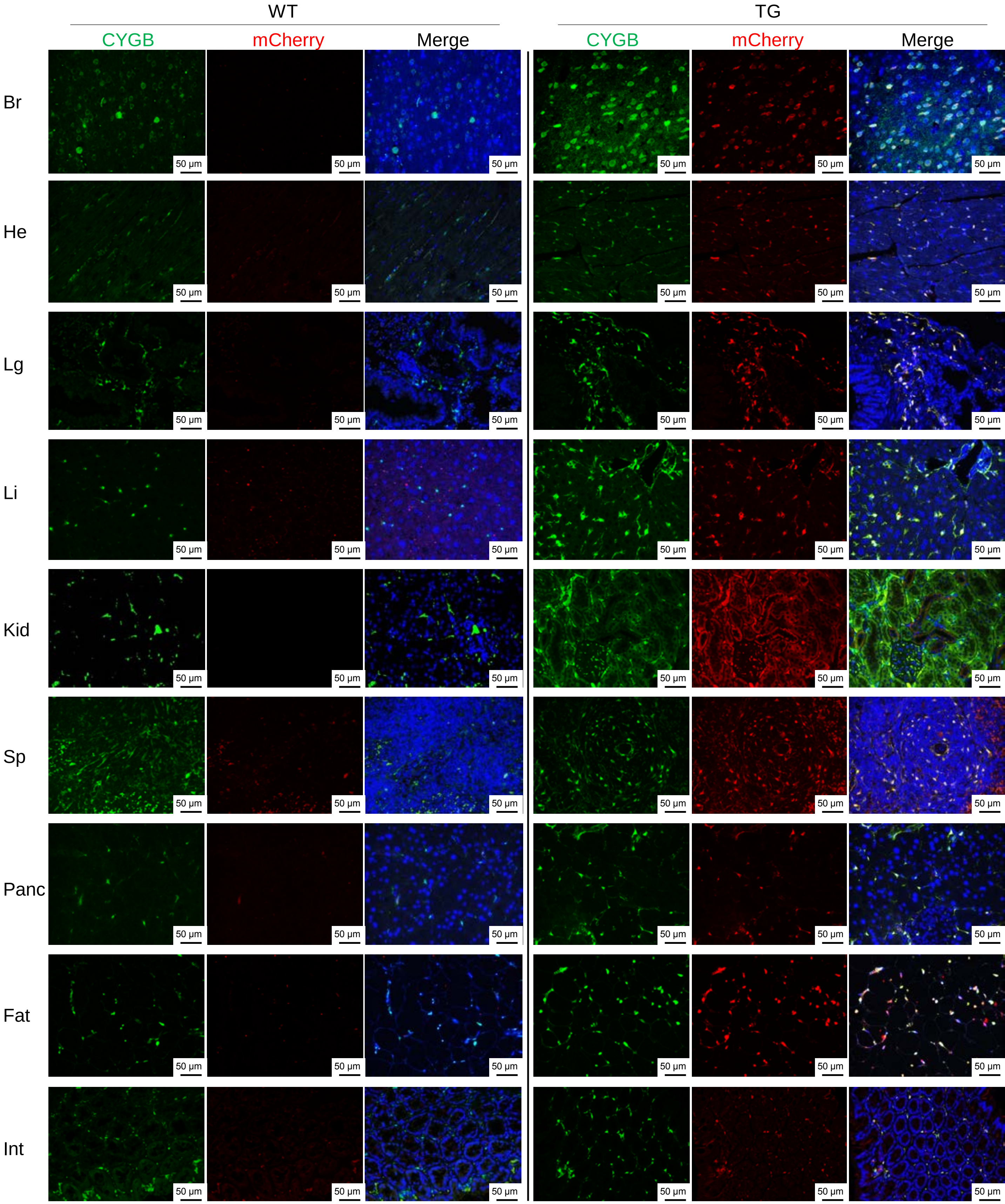


**Supplementary Figure S2.** CYGB (green), mCherry (red), and DAPI (blue) immunofluorescent staining of multi-organs from wild type (WT) and Cygb-TG (TG) mice. *Cygb*-TG mice present much stronger signal of CYGB than WT in all organs (Br, Brain; Li, Liver; Panc, Pancreas; He, Heart; Lg, Lung; Int, Intestine; Sp, Spleen; Kid, Kidney).

# Supplementary Figure S3

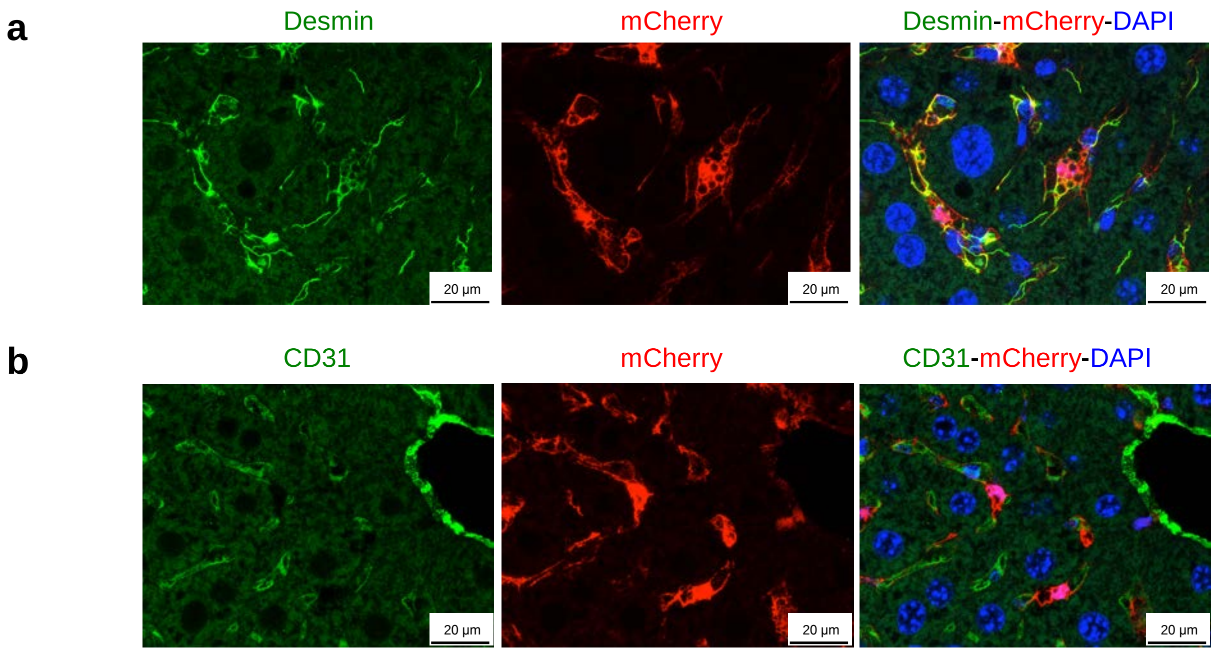


**Supplementary Figure S3.** The representative images of desmin (**a**) or CD31 (**b**) (green), mCherry (red), and DAPI (blue) immunofluorescent co-staining in Cygb-TG livers. mCherry co-localized with desmin as a marker of stellate cells, but not CD31 as a marker of endothelial cells.

# Supplementary Figure S4

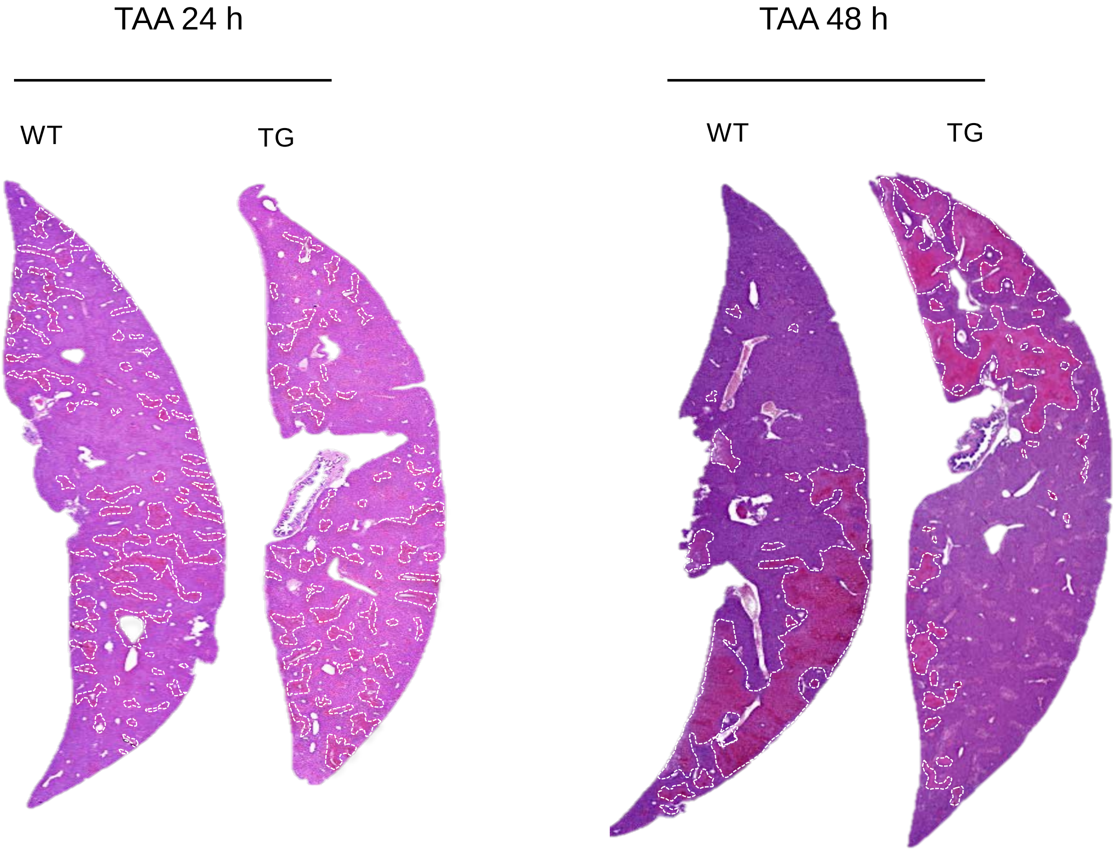


**Supplementary Figure S4.** Histological examination of liver injury from wild type (WT) and Cygb-TG mice (TG) after single dose 50mg/kg TAA administration for 24 hours (24 h) or 48 hours (48 h). Whole lobe images of H&E staining in paraffin-embedded liver sections are shown. Separate images at 100 times magnification were taken by BZ-X700 microscope (Keyence, Osaka, Japan) and merged to the whole lobe pictures by its BZ-X Analyser software. Dash line showed the hemorrhagic area.

# Supplementary Figure S5

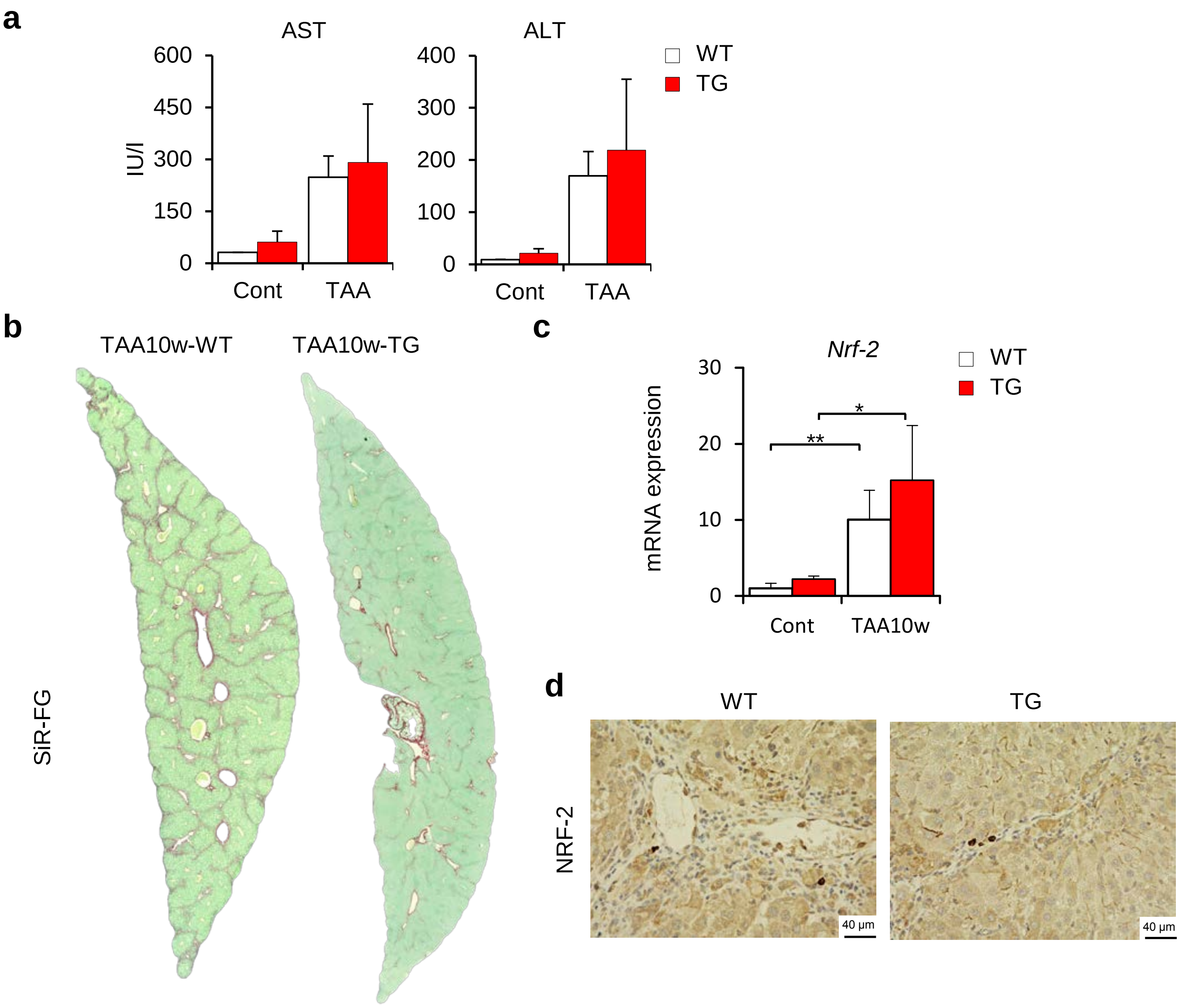


**Supplementary Figure S5**. **a**. Serum levels of AST and ALT activity of wild type (WT) and Cygb-TG mice (TG) were measured after the administration of TAA for 10 weeks. **b.** Sirius red fast green (SiR-FG) staining for collagen deposition in paraffin-embedded liver sections from wild type (WT) and Cygb-TG mice (TG) after 10 weeks TAA treatment are shown. Separate images at 100 times magnification were taken by BZ-X700 microscope (Keyence, Osaka, Japan) and merged to the whole lobe pictures by its BZ-X Analyser software.**c**. mRNA expression of *Nrf-2* (normalized to *Gapdh*, p=0.07) and **d**. NRF-2 immunostaining of livers from wild -type (WT) and *Cygb*-TG mice (TG) after 10weeks TAA treament.

# Supplementary Figure S6

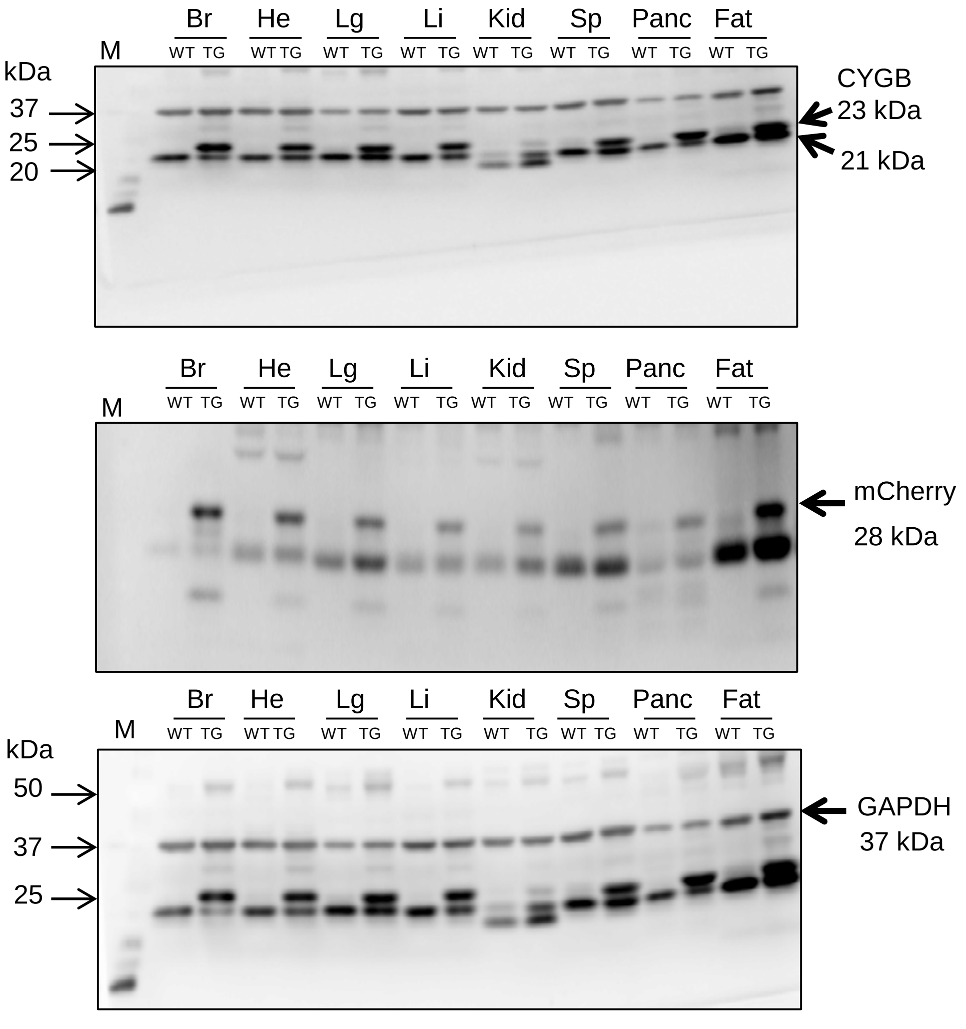


**Supplementary Figure S6.** Expression of CYGB and mCherry in multiple organs of wild-type (WT) and Cygb-TG mice (TG). Full-length Western blots in one gel were performed to examine the CYGB and mCherry expression in WT and Cygb-TG liver (40 µg total protein), which were shown in Figure 1f. n= 3 independent experiments. M, molecular weight marker; Br, Brain; He, Heart; Lg, Lung; Li, Liver; Kid, Kidney; Sp, Spleen; Panc, Pancreas. The images were captured under automatic exposure time or before the bands were saturated.

# Supplementary Figure S7

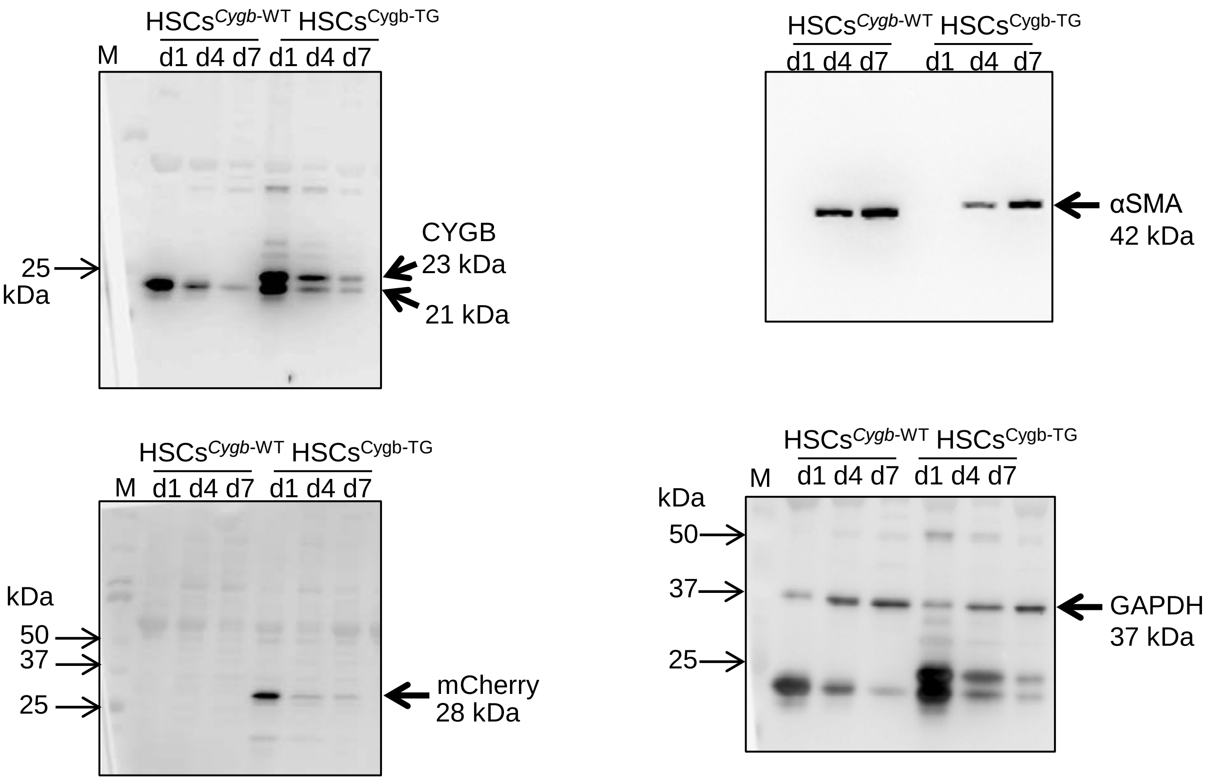


**Supplementary Figure S7:** Expression of CYGB, mCherry and αSMA in primary HSCs$^{Cygb\text{-}WT}$ and HSCs$^{Cygb\text{-}TG}$ in normal conditions at day 1, 4 and 7 culture. Full-length Western blots in one gel were performed using 3.5 μg total protein for each lane, which were shown in Figure 2c. n= 3 independent experiments. M, molecular weight marker. The images were captured under automatic exposure time or before the bands were saturated.

# Supplementary Figure S8

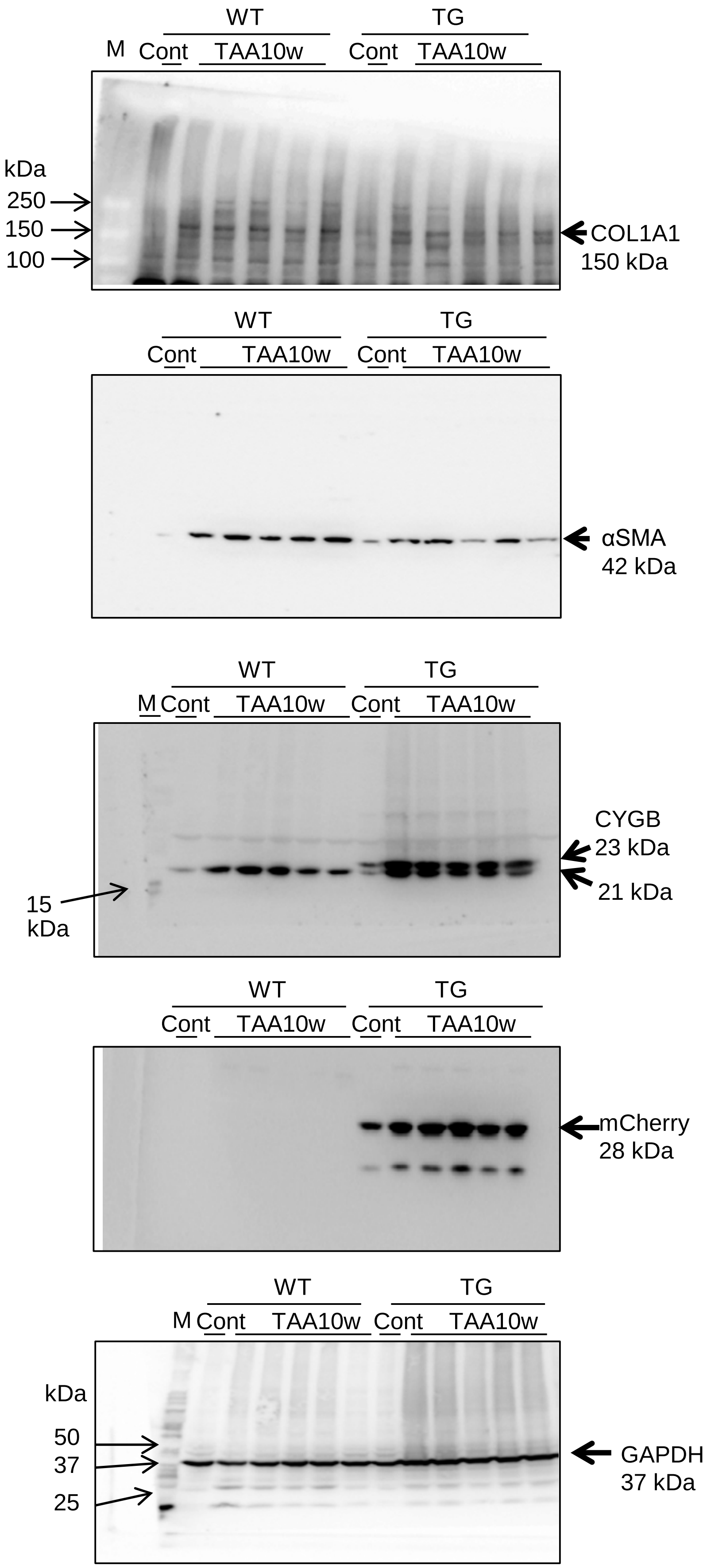


**Supplementary Figure S8.** Full-length Western blots in one gel were performed to examine COL1α1, αSMA, CYGB, mCherry and GAPDH expression from WT and *Cygb*-TG livers (40 µg total protein); which were shown in Figure 4e. n= 3 independent experiments. The images were captured under automatic exposure time or before the bands were saturated.

# Supplementary Figure S9

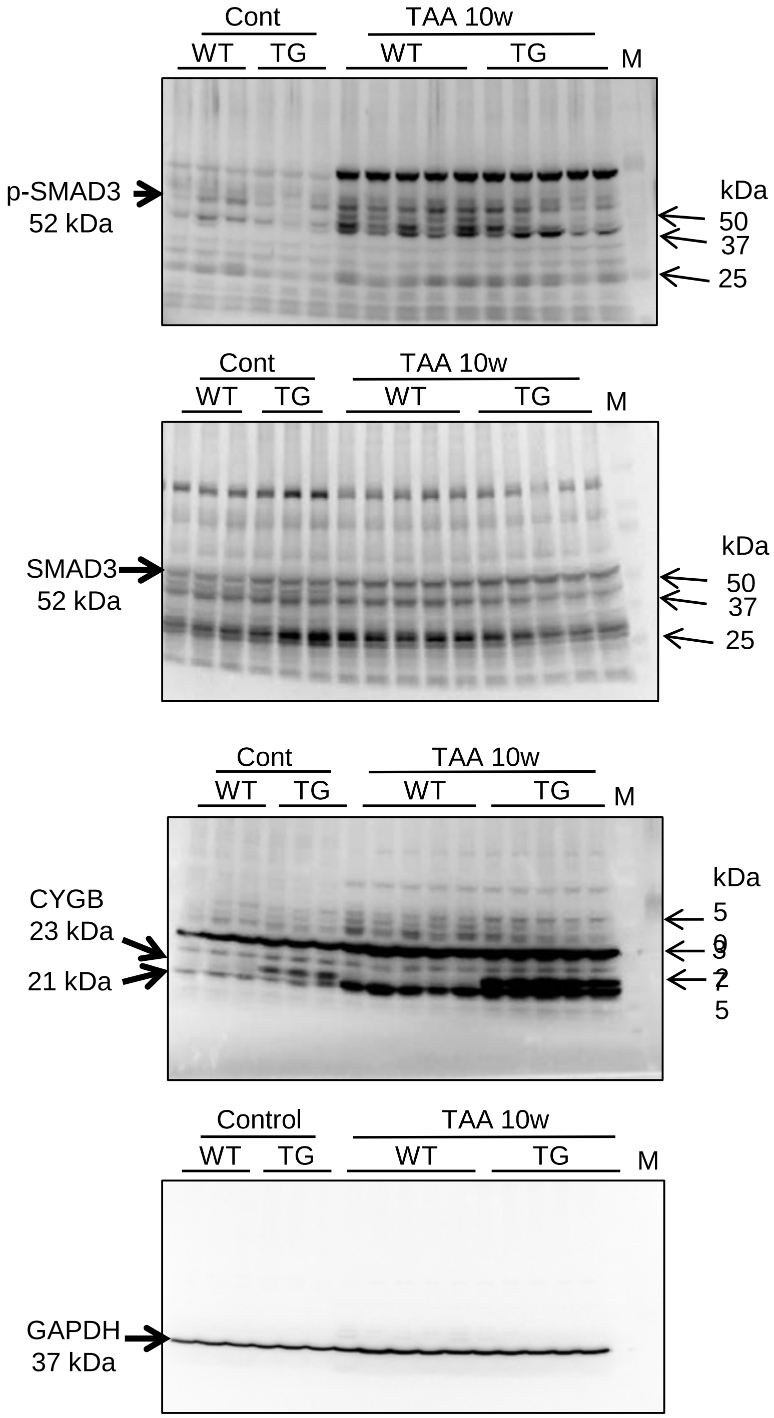


**Supplementary Figure S9.** Full-length Western blots in one gel were performed to examine phosphorylated and total SMAD3, CYGB, GAPDH expression from WT and Cygb-TG livers (40 μg total protein); which were shown in Figure 4g. n= 3 independent experiments. The images were captured under automatic exposure time or before the bands were saturated.

# Supplementary Figure S10

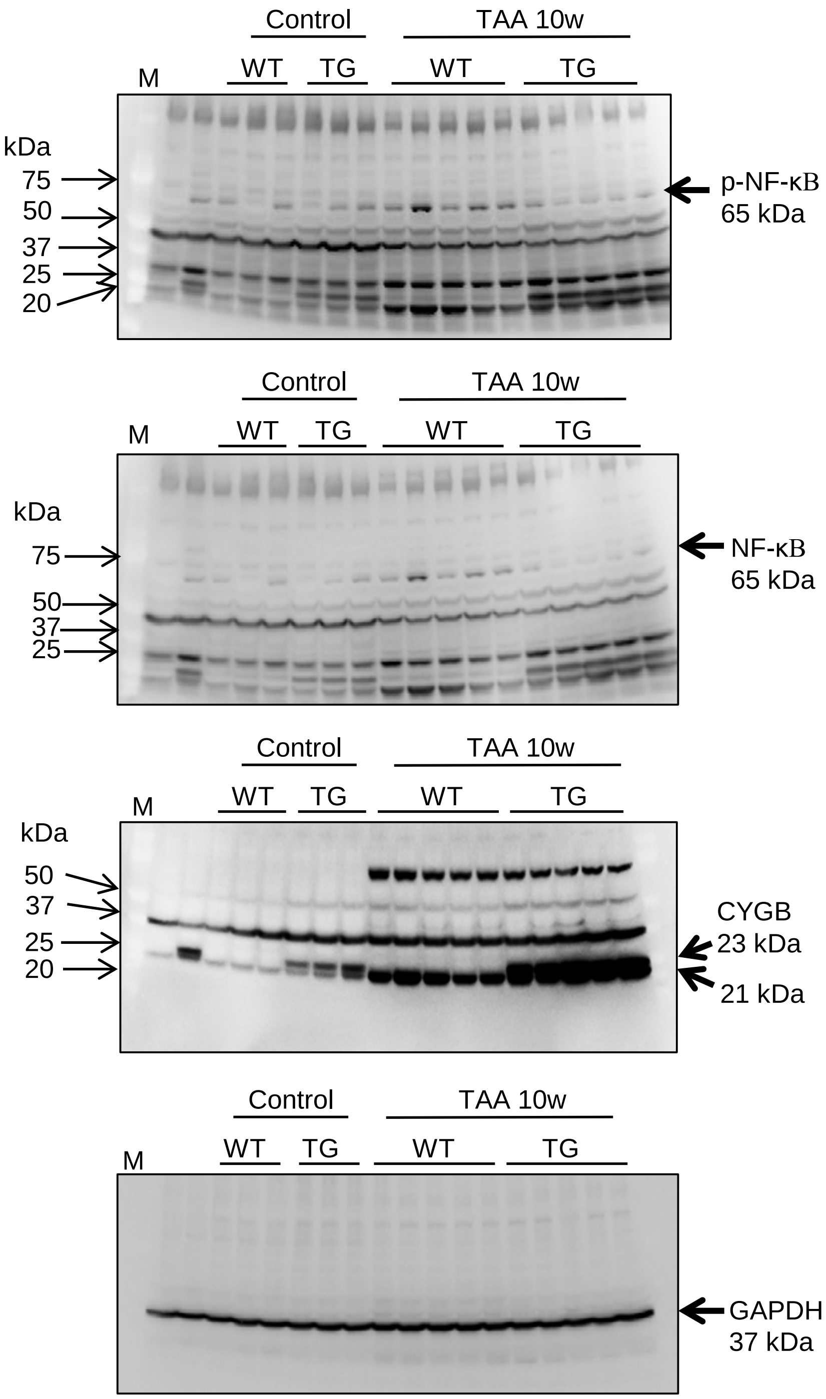


**Supplementary Figure S10.** Immunoblotting for phosphorylated- and total NF-κB in liver tissues from WT and Cygb-TG. Full-length Western blots in one gel were performed to reveal the decrease in phosphorylated- NF-κB expression of Cygb-TG liver (40 μg total protein) compared with WT; which were shown in Figure 5b. n= 3 independent experiments. The images were captured under automatic exposure time or before the bands were saturated.

# Supplementary Figure S11

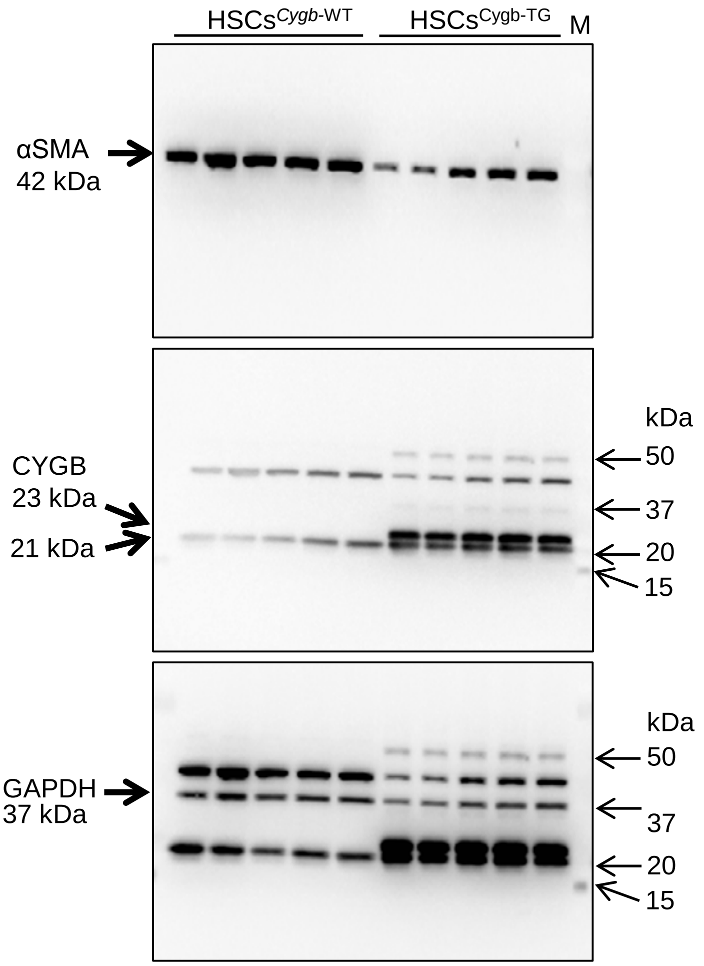


**Supplementary Figure S11.** Overexpression of Cygb alleviated the ROS-induced activation of HSCs. Full-length Western blots in one gel were performed to examine the αSMA, CYGB and GAPDH expression in primary hepatic stellate cells isolated from WT (HSCs$^{Cygb\text{-}WT}$) and Cygb-TG mice (HSCs$^{Cygb\text{-}TG}$) after 48 hours in $H_2O_2$ administration (3.5 μg total protein), which were shown in Figure 6c. n= 3 independent experiments. The images were captured under automatic exposure time or before the bands were saturated.
